# Whole-brain tracking of cocaine and sugar rewards processing

**DOI:** 10.1038/s41398-023-02318-4

**Published:** 2023-01-23

**Authors:** Łukasz Bijoch, Joanna Klos, Monika Pawłowska, Justyna Wiśniewska, Diana Legutko, Urszula Szachowicz, Leszek Kaczmarek, Anna Beroun

**Affiliations:** 1 Laboratory of Neuronal Plasticity, Nencki-EMBL Center of Excellence for Neural Plasticity and Brain Disorders: BRAINCITY, Nencki Institute of Experimental Biology of the Polish Academy of Sciences, Warsaw, Poland; 2 Laboratory of Neurobiology, Nencki-EMBL Center of Excellence for Neural Plasticity and Brain Disorders: BRAINCITY, Nencki Institute of Experimental Biology of the Polish Academy of Sciences, Warsaw, Poland; 3 Institute of Experimental Physics, Faculty of Physics, University of Warsaw, Warsaw, Poland

**Keywords:** Learning and memory, Molecular neuroscience

## Abstract

Natural rewards, such as food, and sex are appetitive stimuli available for animals in their natural environment. Similarly, addictive rewards such as drugs of abuse possess strong, positive valence, but their action relies on their pharmacological properties. Nevertheless, it is believed that both of these kinds of rewards activate similar brain circuitry. The present study aimed to discover which parts of the brain process the experience of natural and addictive rewards. To holistically address this question, we used a single-cell whole-brain imaging approach to find patterns of activation for acute and prolonged sucrose and cocaine exposure. We analyzed almost 400 brain structures and created a brain-wide map of specific, c-Fos-positive neurons engaged by these rewards. Acute but not prolonged sucrose exposure triggered a massive c-Fos expression throughout the brain. Cocaine exposure on the other hand potentiated c-Fos expression with prolonged use, engaging more structures than sucrose treatment. The functional connectivity analysis unraveled an increase in brain modularity after the initial exposure to both types of rewards. This modularity was increased after repeated cocaine, but not sucrose, intake. To check whether discrepancies between the processing of both types of rewards can be found on a cellular level, we further studied the nucleus accumbens, one of the most strongly activated brain structures by both sucrose and cocaine experience. We found a high overlap between natural and addictive rewards on the level of c-Fos expression. Electrophysiological measurements of cellular correlates of synaptic plasticity revealed that natural and addictive rewards alike induce the accumulation of silent synapses. These results strengthen the hypothesis that in the nucleus accumbens drugs of abuse cause maladaptive neuronal plasticity in the circuitry that typically processes natural rewards.

## Introduction

Psychostimulants are drugs that increase the activity of the central nervous system and produce a transient increase in psychomotor action [1]. There is a great variety of such substances, both illicit (i.e. cocaine, methamphetamine) and prescribed (i.e. amphetamine, methylphenidate). Psychostimulants offer a diversity of usage in society, as they increase the performance in cognitive tasks and often serve as treatments for diseases (e.g. attention deficit hyperactivity disorder, excessive sleepiness) [2]. Unfortunately, almost all of them have great addictive potential, and their continued usage leads to progressive dependence. Addiction development is linked to long-lasting, drug-evoked plastic changes. Such plasticity is fundamental for drug-associated memories and is believed to be crucial for understanding addiction development [3].

It is hypothesized that psychostimulants tap into neuronal circuitry usually processing pleasant, non-addictive experiences driven by natural rewards [4]. Thus, to understand the salience of addictive substances, one shall consider the natural reward-processing pathways. However, a detailed comparative analysis of addictive and natural reward exposure has not yet been done. Such evaluation would first require imaging of the reward effects on a brain-wide scale. Until recently, whole-brain imaging in human subjects was the only way to look at neuronal network activity holistically. However, since psychostimulants are highly addictive, such studies on human subjects would be controversial. Recently, thanks to modern imaging techniques, such screening studies on animals have been made possible. Specifically, brain-wide imaging of the cleared rodent brain, with a prominent high throughput and cellular spatial resolution [5] proved very useful in whole-brain neuronal profiling and tracing of neuronal projections [6]. To date, the whole-brain imaging approach on cleared samples has successfully been applied in studying various forms of learning including fear conditioning [7–9], food craving [10], alcohol addiction models [11], exposure to opioids [12], and psychostimulants such as cocaine [13, 14].

Thus, in this study we used whole-brain imaging of activity-dependent plasticity marker c-Fos in mice, to obtain insight into neurocircuits recruited by either addictive or natural rewards. We examined brain changes after the acute (single) and prolonged (7 days) exposure to sucrose self-administration and cocaine i.p. injections, by creating a map of reward-processing brain regions to later holistically look at the functional connectivity of these neuronal networks. We found that while initially, the effect of both types of rewards was similar, after prolonged exposure, cocaine but not sucrose, caused a sustained reorganization of the brain modularity. We further characterized the cocaine- or sucrose-related exposure changes on the cellular level in the nucleus accumbens (ACB), a structure strongly activated by both types of rewards. With electrophysiology recordings, we found that both addictive and natural rewards triggered plastic changes, manifested by the generation of silent synapses.

## Materials and methods

### Animals and behavioral training

All experiments were performed on BAC, a double-transgenic mouse line, obtained by crossing B6.Cg-Tg(Drd1a-tdTomato)6Calak/J (D1-TdTomato) mice with Tg(Drd2-EGFP)S118Gsat/Mmnc (D2-GFP) mice. Animals of both sexes, 3–5 months old were housed in individual cages under a 12 h light/12 h dark cycle with food and water *ad libitum*. All studies were performed under the European Communities Council Directive of November 24, 1986 (86/609/EEC), Animal Protection Act of Poland, and approved by the 1^st^ Local Ethics Committee in Warsaw (permission number 463/2017). All efforts were made to minimize the number of used animals and their suffering. Numbers of animals in each group were estimated based on published research: Beroun et al. 2018 and Shukla et al. 2017 for electrophysiological recordings and Kimbrough et al. 2021 for iDisco clearing and light-sheet imaging.

For the natural reward exposure mice received an additional bottle filled with either sweet (7.5% sucrose solution) or fresh water for 2 h during the light phase of the day. During the experiment, a group of randomly selected 9 mice was moved to another cage to measure the preference of mice for sweet water. In this cage, bottles were connected with an external lickometer based on the microcontroller Arduino Leonardo, which detects the electric circuit’s closing and thus allows one to determine the time in which an animal is drinking from a bottle (Fig. S1A, B). The data was streamed onto a hard drive with CoolTerm 1.9.0 software. Preference was calculated as time spent drinking from the left or right bottle over the total time spent on drinking from both bottles in 2 h.

For the pharmacological reward exposure, mice were injected with 100 μl of saline or 100 μl of 20 mg/kg of cocaine hydrochloride dissolved in saline. A group of randomly selected mice (11 for saline and 13 for cocaine i.p. injection) during the whole experiment, immediately after the injection was placed in another cage and their locomotor activity was recorded with a camera for 30 min (Fig. S1C). Bonsai software was used to analyze the locomotor activity of these mice [15]. The threshold for distinguishing the mouse from the background was determined manually. It allowed us to create a mask of the mouse and its center (centroid) was used to later calculate the movement of a mouse according to the equation for the Euclidean distance:

$d\left( {p,q} \right) = \sqrt {\left( {q_1 - p_1} \right)^2 + \left( {q_2 - p_2} \right)^2}$, where (*p*_1_, *p*_2_) and (*q*_1_, *q*_2_) are coordinates of the centroid at subsequent time points.

### Optical tissue clearing and whole-brain c-Fos imaging

An updated protocol from http://idisco.info was used for the whole-brain clearing technique [16, 17]. Two hours after the last reward exposure, mice were perfused with PBS enriched with heparin (for clearance of the blood) for 10 min and then with 4% paraformaldehyde for 5 min. Brains were extracted and cut in half. An intact hemisphere was used for the standard three-week-long iDisco + protocol with the use of antibodies against c-Fos (Synaptic Systems 226 003, 1:2000) and complementary secondary antibody conjugated with Alexa Fluor 647. Briefly, each sample underwent a dehydration procedure with methanol and dichloromethane washing. Next, samples were bleached with H_2_O_2_ and rehydrated again with decreasing concentrations of methanol. Next, during immunolabeling steps, samples were permeabilized with TritonX-100 solution, washed with blocking buffer, and incubated with primary antibodies (rabbit, anti-c-Fos: Synaptic Systems 226 003, 1:2000), and then secondary antibodies (goat, anti-rabbit with Alexa Fluor 647: ThermoFisher (Invitrogen) A32733, 1:2000). Following recommendations given by Renier et al., 2016 secondary antibody was centrifuged at 20,000 g for 10 min to prevent the formation of precipitates. During the clearing procedure, samples were dehydrated again with methanol and dichloromethane solutions, and finally, they were incubated in DiBenzyl Ether (DBE). Also, to prevent tissue damage, exchanging methanol solutions was done rapidly to prevent the drying of the tissue. At each step, to prevent oxidation, special care was taken to completely fill 5 ml Eppendorf tubes with appropriate liquids and solutions. After clearing, brains were left in DBE for at least 2 days at 4 °C for fluorescence stabilization. Cleared samples were imaged sagittally, using a custom-made light-sheet microscope with two Nikon 4X-PF dry objectives for illuminating and multi-immersion LaVisionBioTec 4x NA 0,5, WD 6 mm for imaging. Lasers with wavelengths of 488 nm and 638 nm were used to excite the tissue fluorescence and c-Fos signal respectively. The light-sheet thickness was 10 μm. To avoid distortions at the edges of the field of view (caused by the divergence of the stationary light sheet), ROI size was limited to an area where the axial resolution was uniform. The voxel size of acquired images for autofluorescence was 1.45 × 1.45 × 5 μm and 1.45 × 1.45 × 10 μm for the c-Fos signal. As described previously by Renier et al., 2014, the clearing procedure completely abolished the endogenous fluorescence coming from TdTomato and GFP expressed from D1 and D2 promoters. Even with 488 nm illumination (wavelength corresponding to GFP excitation), D2 cells were not visible. Thus, we assumed that the whole signal collected with 638 nm light was corresponding to the c-Fos signal (for a detailed description of the microscopic setup see [18, 19]).

### Computational analysis of cleared brain

As specimens were larger than the field of view of the microscope, the imaging was performed as a series of 3D tiles that were subsequently stitched, fused, and exported as a single tiff stack using Bigstitcher Fiji plugins [20].

For c-Fos detection, the automated pipeline ClearMap was used [16]. For a few structures, ClearMap failed to properly generate the data: Interfascicular nucleus raphe (IF), oculomotor nucleus (III), subfornical organ (SFO), rhomboid nucleus (RH), interanteromedial nucleus of the thalamus (IAM), induseum griseum (IG), magnocellular reticular nucleus (MARN), accessory facial motor nucleus (ACVII), facial motor nucleus (VII), septohippocampal nucleus (SH), infralimbic area, layer 1 (ILA1), agranular insular area, dorsal part layers 1–6b (AId6b, AId6a, AId5, AId2/3, AId1). For most of these structures, the detection failure was due to their small size and location at the edge of the tissue sample (Fig. S4). The exception was the dorsal part of the agranular insular area located in the cortex. Numbers of c-Fos-positive cells, as well as the c-Fos density values for individual mice, are available at https://github.com/BijochLukasz/Whole-brain-tracking-of-cocaine-and-sugar-rewards-processing.

In the analysis of the fold increase of c-Fos level, behavioral groups are normalized to home cage data. Individual values indicate the quotient of the c-Fos level after reward treatment divided by its level in corresponding control groups.

Identification of structure co-activation and hierarchical clustering was performed using RStudio software. Separate Pearson correlations were calculated across each group to compare c-Fos data from each brain region to each of the other brain regions. Data were arranged into anatomical groups based on the Allen Mouse Brain Atlas. The total Euclidean distance between structures, which is a square root of the squared differences between structures, was calculated for the hierarchical clustering of each group separately. Then, the order of structures was rearranged for each group based on hierarchical clustering to finally merge groups back together into a list of structures. The correlation or distance heat maps were then applied to all treatments [21]. RStudio packages pheatmap and dendextend were used for heatmap generation, clustering, and modularity analysis. For scripts see: https://github.com/BijochLukasz/Whole-brain-tracking-of-cocaine-and-sugar-rewards-processing.

A network graph (Fig. S5) was created based on regions activated by reward treatment with the highest probability (*p*-value < 0.01 cut-off after Benjamini-Hochberg’s false discovery correction; see statistical analysis). These structures are referred to later as nodes of the created graph network. Results from separate Pearson correlations calculated across these structures were used to determine connections between them (*r* > 0.75; positive correlations later referred to as connections between nodes—edges). For graph network visualization we used a pyvis library for Python by West Health Institute. The position of elements on the graph was determined with the model simulating physics, based on the repulsion between edges (hierarchical repulsion model). All nodes and numbers of their edges are available in the Additional Fig. 1 at: https://github.com/BijochLukasz/Whole-brain-tracking-of-cocaine-and-sugar-rewards-processing.

**Fig. 1:**
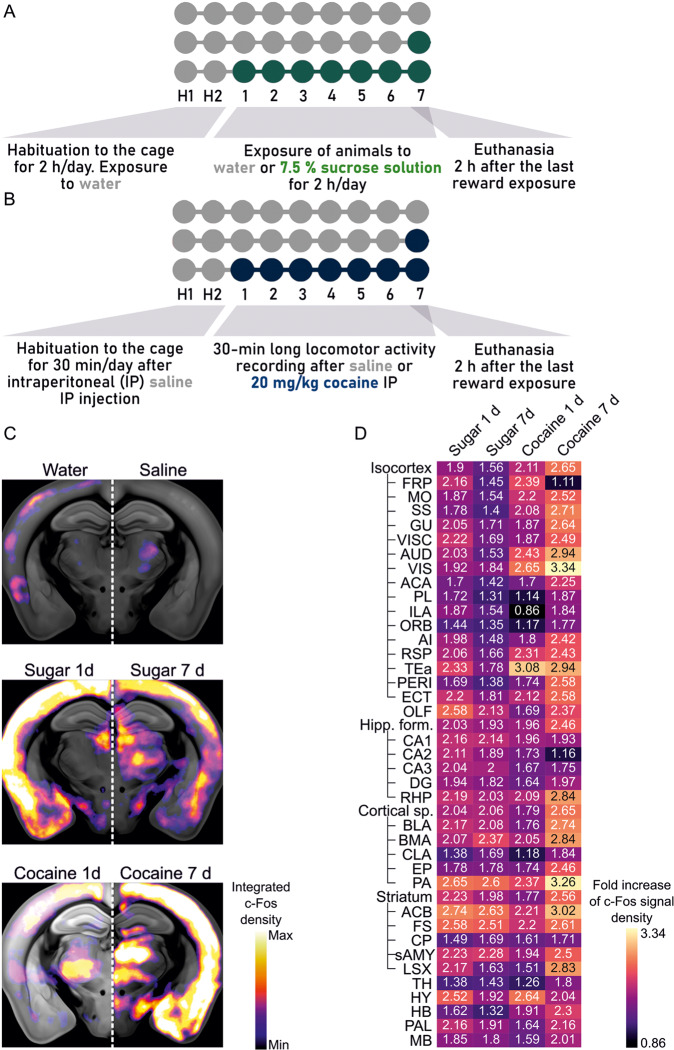
Design of the behavioral procedure and c-Fos mapping in the brain. **A**, **B** Behavioral paradigm for sucrose (**A**) and cocaine exposure (**B**). **C** Heatmaps of the c-Fos density averaged from mice exposed to natural and addictive rewards. The warmer color, the more robust c-Fos expression in the brain region. **D** Fold increase of the c-Fos signal density, where the reward-treated groups are normalized to controls (either water- or saline-treated groups). Frontal pole (FRP), somatomotor areas (MO), somatosensory areas (SS), gustatory areas (GU), visceral area (VISC), auditory areas (AUD), visual areas (VIS), anterior cingulate area (ACA), prelimbic area (PL), infralimbic area (ILA), orbital area (ORB), agranular insular area (AI), retrosplenial area (RSP), temporal association area (Tea), perirhinal area (PERI), ectorhinal area (ECT), olfactory areas (OLF), hippocampal formation (Hipp f.), hippocampal CA1 (CA1), hippocampal CA2 (CA2), hippocampal CA3 (CA3), dentate gyrus (DG), retrohippocampal region (RHP), cortical subplate (Cortical sp.), basolateral amygdala (BLA), basomedial amygdalar nucleus (BMA), claustrum (CLA), endopiriform nucleus (EP), posterior amygdalar nucleus (PA), nucleus accumbens (ACB), fundus of striatum (FS), caudoputamen (CP), striatum-like amygdalar nuclei (sAMY), lateral septal complex (LSX), thalamus (TH), hypothalamus (HY), hindbrain (HB), pallidum (PAL), midbrain (MB).

Fold indexes were calculated with the following equation: ${{{\mathrm{Fold}}}}\,{{{\mathrm{index}}}} = \frac{{\bar \mu _{{{{\mathrm{reward}}}}}}}{{\bar \mu _{{{{\mathrm{control}}}}}}}$, where μ is the number of activated neurons for individual behavioral groups.

### Electrophysiology recordings

The whole-cell patch-clamp technique was used to measure the percentage of silent synapses and decay time of NMDAR-mediated currents. Two hours after the beginning of the reward exposure session, mice were anesthetized with isoflurane and decapitated. Their brains were removed and cut into 250 μm coronal slices using Leica VT 1200 S vibratome in ice-cold NMDG solution (135 mM NMDG, 1.2 mM KH_2_PO_4_, 1 mM KCl, 1.5 mM MgCl_2_, 0.5 mM CaCl_2_, 20 mM choline bicarbonate, 10 mM D-glucose, saturated with carbogen. Next, slices were incubated for 10 min in a 31 °C water bath). Then, slices were kept at room temperature in artificial cerebrospinal fluid solution (ACSF 119 mM NaCl, 2.5 mM KCl, 1.3 mM MgCl_2_, 1 mM NaH_2_PO_4_, NaHCO_3_, 20 mM D-glucose, 2.5 mM CaCl_2_ saturated with carbogen) for at least 1 h before the start of the recording. To patch neurons, we used borosilicate glass capillaries (of 4–6 MΩ resistance) filled with internal solution (130 mM Cs-gluconate, 20 mM HEPES, 3 mM TEA-Cl, 0.4 mM EGTA, 4 mM Na_2_ATP, 0.3 mM NaGTP, 4 mM QX-314Cl with pH: 7.0-7.1 and osmolarity: 290–295 mOsm). Data was acquired with Igor Pro (Wavemetrics) software with an NPI amplifier and digitized at 10 kHz with ITC-18 InstruTECH/HEKA. Recorded currents were filtered at 2 kHz. Series and input resistances were monitored during recordings.

For the percentage of silent synapses, the minimal stimulation protocol was used [22]. Thus, the strength of the electric pulse for stimulation was adjusted to obtain both responses and failures of AMPAR- and NMDAR-mediated currents, recorded at -60 and + 45 mV respectively. Successes and failures were distinguished visually. The percentage of silent synapses was calculated with the equation: $\% \,silent\,synapses = 1 - \frac{{ln_{F - 60mV}}}{{ln_{F + 45mV}}}$, where F-60 mV and F + 45 mV are failure rates at -60 mV and + 45 mV respectively. Therefore, the higher ratio of failures recorded at F-60 mV over failures recorded at F + 45 mV corresponds to the higher percentage of silent synapses. For measuring the decay time of NMDAR-mediated responses, excitatory postsynaptic currents, measured at+45 mV were recorded and analyzed using Clampfit 10.3 software.

D1 and D2 cells of the ACB shell were distinguished visually based on their fluorescence (green for D2 and red for D1).

### Immunohistochemistry and imaging of brain slices

2 h after the last reward exposure mice were perfused with PBS enriched with heparin (for clearance of the blood) for 10 min and then with 4% PFA in PBS for 5 min. Brains were kept in PFA at 4 °C overnight and then cut into 70 μm coronal slices on vibratome Leica VT 1000 S.

For c-Fos labeling, slices were washed 4x for 5 min with PBS and then 1x for 10 min with 0.25% Triton X-100 in PBS at RT. Next in 0.1% Triton X-100, 3% bovine serum albumin (BSA) in PBS for 3 h. Then, slices were washed 1x for 5 min with PBS and incubated with a mix of primary antibodies (against c-Fos (Synaptic Systems 226 003, 1:2000); against mCherry (ThermoFisher M11217, 1:1500); against GFP (Abcam, ab5450, 1:1500)) dissolved in 0.1% Triton X-100, 0.1% BSA in PBS in 4 °C overnight. Next, slices were washed 4x for 5 min with PBS and incubated for 3 h with a mix of secondary antibodies dissolved in PBS. Slices were mounted on microscope slides and imaged with a confocal microscope Inverted Axio Observer Z.1 (with EC Plan-Neuofluar 10x/0.30 dry objective).

With the use of Fiji software, c-Fos positive nuclei were counted manually. All experiments were done with distinguishing D1 and D2 cells. Previous experiments have shown that these two populations are highly non-overlapping in ACB. The presence and absence of fluorescence signals were used to predict D1 versus D2 cells [23]. Similarly, in our studies, in some cases, where only one population was fluorescently labeled, cells without fluorescence were counted as the other population.

### Statistical analysis

For analyzing the whole-brain data R script written by us, statistic*_*binomial.Red was used. First, the generalized linear model (GLM) was fitted to the number of detected c-Fos positive cells in each brain region in every animal group using a negative binomial to model data points’ distribution. Next, Dunnett’s correction was performed. We compared a high number of structures, so to avoid false-positive discoveries, Benjamini-Hochberg’s false discovery rate was performed on *p*-values with a cut-off of 0.1 for the number of structures with elevated c-Fos levels after natural or addictive treatment [24]. For graph analysis, we used stricter statistics and the *p*-value cut-off was set at 0.01. For the data from immunohistochemistry on brain slices and electrophysiology, statistical analysis was performed using GraphPad Prism software. The distributions of datasets were evaluated with the Shapiro-Wilk normality test. Then the two-way ANOVA test was performed. The number of mice/slices/neurons and statistical significance (when *p* < 0.05) are reported below the figures. All error bars represent s.e.m. values.

## Results

### Overall description of the behavioral model

For all experiments, we used transgenic mouse lines with fluorescently labeled dopamine-sensitive neurons (D1 cells with *Drd1a* promoter-driven tdTomato expression and D2 cells with *Drd2*-driven GFP expression).

For natural reward exposure, mice received an additional bottle in the cage for 2 h/day. The bottle was filled either with water (control group) or 7.5% sucrose solution (Fig. 1A). Mice were exposed to sweet water either once, during the last day of the experiment, or for 7 consecutive days (sugar 1 d or sugar 7 d groups respectively). Mice exhibited a profound preference for a bottle filled with sweet water during the entire duration of the experiment (Fig. S1A, B).

As a pharmacological reward that carries a strong addictive potential, we used intraperitoneal injections (i.p.) of 20 mg/kg of cocaine solution (or saline in the control group, Fig. 1B). Mice received cocaine injection only on the last day of the experiment or for 7 consecutive days (cocaine 1 d or cocaine 7 d groups respectively). Repetitive cocaine injections induced increased locomotor activity in the form of locomotor sensitization, i.e. each consecutive cocaine injection had a more substantial behavioral effect (Fig. S1C).

Two hours after the last extra-bottle exposure or i.p. injection mice were sacrificed and their brains were used for further studies.

### c-Fos expression triggered by sweet water and cocaine

To examine which parts of the brain are activated by natural and addictive rewards, we used a whole-brain imaging approach with tissue clearing combined with immunohistochemistry (iDisco+) and light-sheet microscopy [17]. To mark cells activated by reward exposure, we holistically labeled the product of one of the IEGs, *c-fos*. c-Fos is a transcription factor widely used as a marker of neuronal activation that mediates plasticity at the cellular, synaptic, and network levels [25–27]. Notably, a c-Fos expression typically follows a time-specific course, in which protein is detectable in neuronal nuclei within 1–3 h after induction [25].

Optically cleared and immunolabelled samples were imaged with a self-made light-sheet microscope [19] and images of the brain were aligned to the Allen Brain Atlas using the ClearMap software [16]. The number of c-Fos + nuclei was automatically detected and associated with specific brain regions (Fig. 1C). Finally, the numbers of c-Fos + corresponding to each structure were divided by the volume of these structures to transform the data into the signal density. Altogether, we analyzed 382 structures, which were classified according to the Allen Brain Atlas. For each structure, we calculated the fold increase of c-Fos, where behavioral groups were normalized to control groups (Fig. 1D). This brief analysis showed the vast activation throughout the brain.

To discover brain regions activated by rewards we compared c-Fos signal density for each structure in sucrose- or cocaine-treated groups with their respective controls. Thus, we used the generalized linear model (GLM) followed by Dunnett’s correction and Benjamini-Hochberg’s correction to avoid false-positive results (type I errors) from a huge amount of data. The created list of activated structures is supposed to act as a guideline for further, more detailed studies on the processing of different rewards. Therefore, to avoid the risk of false negative errors (or type II errors) we used the corrected *p*-value with a cut-off of 0.1 as statistically-relevant changes. The complete hierarchical list of analyzed structures (and corresponding abbreviations) with mean values of c-Fos density and statistical significances for differences between groups after correction is presented in Fig. S3.

**Fig. 2:**
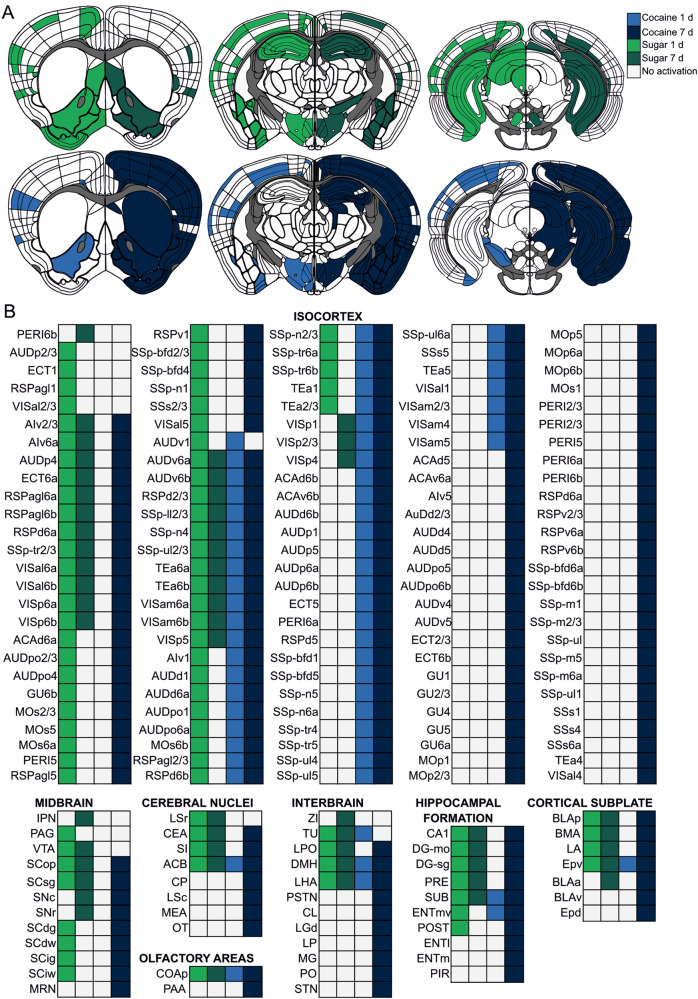
c-Fos expression mapped in the reward system of the brain. **A** Brain schemes based on Allen Brain Atlas with colored structures with significantly elevated c-Fos levels. **B** List of brain structures from schemes in **A** with significantly elevated c-Fos levels. N Water = 7, N Sugar 1 d = 5, N Sugar 7 d = 7, N Saline = 7, N Cocaine 1 *d* = 6, N Cocaine 7 d = 6.

**Fig. 3:**
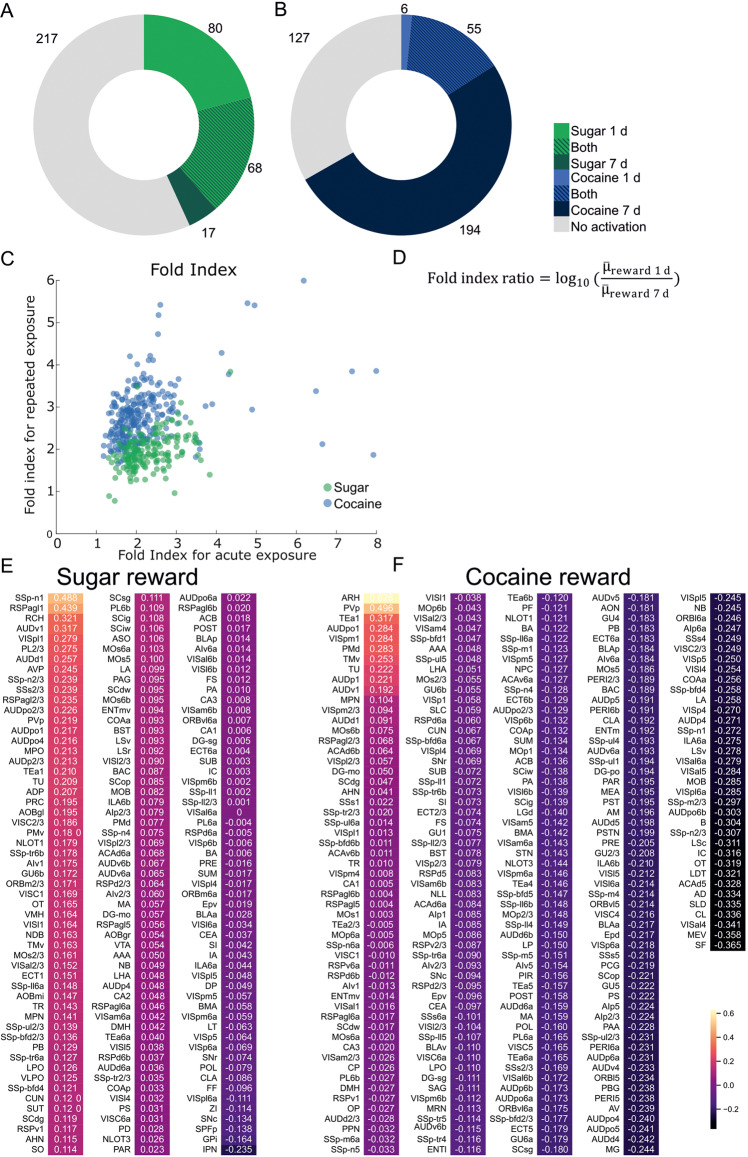
c-Fos expression in the brain after acute and prolonged exposure to reward. **A**, **B** Number of structures with elevated c-Fos level after natural (**A**) or pharmacological reward (**B**) treatment. **C** Graph shows fold indices for structures after either acute or prolonged exposure to either natural (green) or pharmacological (blue) reward exposure. **D** Fold index ratio equation used to compare effects of acute and prolonged reward exposure. **E**, **F** Individual fold indexes ratios for natural (**E**) and pharmacological (**F**) rewards. Fold index ratios were calculated for structures, which compared to control groups had a *p*-value < 0.1 either for 1 day or 7 days of reward treatment. For each structure *p*-value was calculated. First, a generalized linear model (GLM) was calculated. For each GLM a Dunnett’s test was performed. Finally, due to a large number of structures a Benjamin-Hochberg false discovery rate correction was performed on *p*-values with a 0.1 cut-off. N Water = 7, N Sugar 1 d = 5, N Sugar 7 d = 7, N Saline = 7, N Cocaine 1 d = 6, N Cocaine 7 d = 6.

Both single and repeated exposure to rewards caused massive cell activation in distant parts of the mouse brain. We found structures highly activated only by cocaine (e.g. perirhinal cortex) or sugar (lateral septal nucleus (LSr), ventral tegmental area (VTA)) or by both types of rewards (e.g., nucleus accumbens (ACB); Fig. 2A, B; Fig. S3). Altogether, a single sugar exposure resulted in a higher number of activated structures compared to repeated sucrose treatment. In contrast, the acute and prolonged cocaine treatment caused the opposite pattern of brain activation. Here, 7 days of cocaine injections elevated c-Fos levels in more brain regions than a single dose of cocaine (Fig. 3A, B). This prolonged cocaine exposure caused excessive brain activation, which covered the majority of studied structures (Fig. 2A, B; Fig. 3). To further compare the effects of acute and prolonged reward exposure we compared fold indices for particular structures (Fig. 3C, E, F). Notably, the higher number of activated structures was associated with the higher number of c-Fos positive cells per particular structure.

### Altered co-activation and modularity of brains after reward treatments

Next, we examined whether cocaine and sucrose exposure alters the global organization pattern of neuronal populations. For that purpose, we used functional connectivity analysis to identify connections between brain regions based on statistical, linear correlations of their c-Fos expression, thus highlighting which structures share the most and least similar pattern of c-Fos density changes. This approach allows us to determine the modularity of the brain that quantifies the extent to which a network is divided into distinct subnetworks unitedly processing a task [28]. Modularity is also referred to as a biomarker of global neuronal reorganization and it was previously used to determine the large-scale changes in functional neural co-activation caused by alcohol abstinence and exposure [11, 21].

To inspect the differences in brain connectivity between the groups, we first visualized the interregional c-Fos correlation for each group separately, creating correlation matrices (Fig. S2). These correlation matrices were organized according to traditional anatomical groups from the Allen Mouse Brain Atlas. Overall, reward-treated groups exhibited a lower cross-correlation level between brain regions than in control conditions. This is expected, as water and saline treatments resulted in a minimal increase in c-Fos expression, thus exhibiting a very similar pattern throughout the whole brain, hence leading to a strong correlation of such minimal signal (see Fig. 1C). Individual clusters of anticorrelated structures were distinguishable in the thalamus and anterior cingulate area (isocortex) of mice with prolonged natural reward exposure and the thalamus of mice with acute cocaine treatment. Notably, acute sugar and prolonged cocaine treatments exhibited a widespread pattern of anticorrelated structures in the whole brain. Finally, well-recognizable anticorrelated structures were found in the hypothalamus of mice exposed to water (both fresh and sweet water) but not in mice after cocaine injections.

**Fig. 4:**
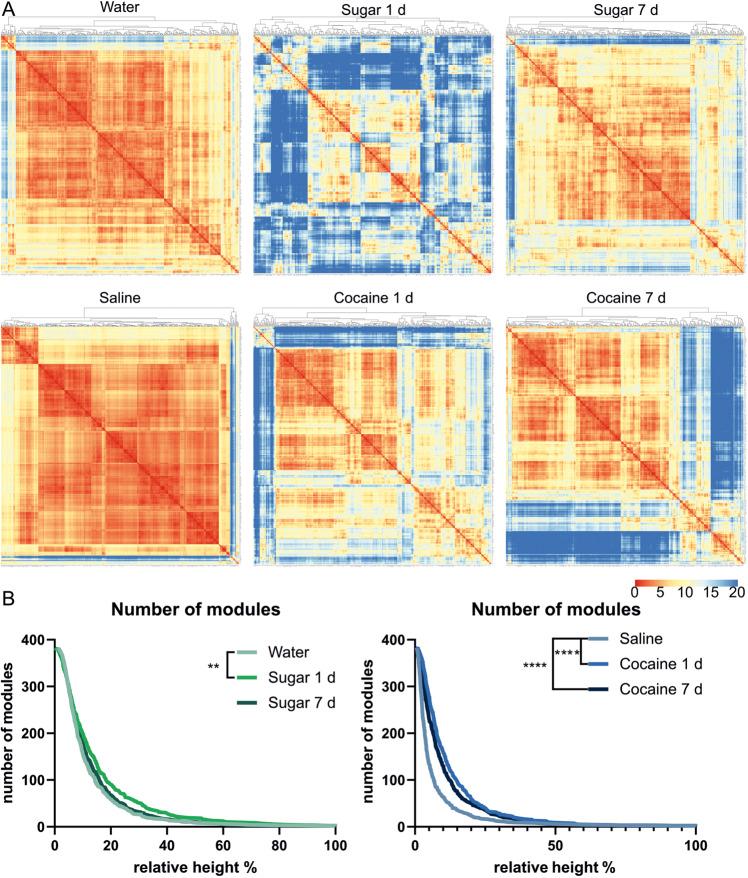
Global pattern of the reorganization of the brain activity after sugar or cocaine exposure. **A** Hierarchical organization of sub-networks of brains of mice exposed to natural and pharmacological rewards. The Euclidean distance between brain structures was calculated to find modules of similar brain structures. Graphs showing sub networks among brain structures created based on the distance between brain regions. Warmer colors indicate a shorter Euclidean distance, indicating that two structures have similar c -Fos signal density correlation patterns with other structures. On the top of each graph are dendrograms created based on Euclidean distances between structures (indicating the similarity of co-activation between structures). **B** Number of modules per group based on cutting the dendrograms at different percentages of tree heights (indicating different similarities between co-activated structures). Statistical difference was calculated with the Mantel-Cox test; Saline vs. Cocaine 1 d and Saline vs. Cocaine 7 d ****=*p* < 0.0001; Water vs. Sugar 1 d **=*p* = 0.0013; Water vs. Sugar 7 d *p* = 0.2426.

Next, regions with a similar co-activation profile across all other brain regions were grouped into modules using hierarchical clustering (Fig. 4A). This allowed us to compare the brain modularity of the studied groups. For this, we plotted the number of clusters to the percentage of similarity across co-activated structures (relative height of created dendrograms; Fig. 4B). We found increased modularity after single, but not after prolonged exposure to sugar (Fig. 4B). Conversely, in cocaine-treated groups modularity was increased after both single and repeated exposure to reward.

### Synaptic plasticity in the nucleus accumbens after reward treatments

Given the profound differences in reward processing observed thus far using our holistic approach, we focused on characterizing the effects of different rewards on a cellular level. To select a candidate structure for this analysis we focused only on brain regions with the most significant difference (*p*-value < 0.01) in activation compared to control groups. As only a few structures passed this cut-off in the acute cocaine exposure group, for detailed comparison we focused only on groups exposed to rewards for a prolonged time. We found 22 such structures in the group treated with sugar for 7 days and 80 in the cocaine-treated group. Only four of these brain regions were common for natural- and addictive-reward treatments: the fundus of the striatum (FS), the nucleus accumbens (ACB), the primary visual area layer 6a (VISp6a), and the primary auditory area layer 4 (AUDp4). To find the best candidate among these four regions, we analyzed correlations between 22 sucrose-activated structures and between 80 cocaine-activated structures. Then, we visualized them in a graph network with connections between positively correlated structures (Fig. S5A, B). FS, ACB, and VISp6a appeared to be central hubs in the network, highly connected with other structures. For further characterization of sucrose and cocaine involvement on a molecular level, we chose one of these regions, the ACB. The ACB is a brain structure responsible for the execution of motivated behaviors, deeply involved in reward processing and the development of addiction [29]. It is mainly composed of two highly non-overlapping populations of dopamine-sensitive medium-spiny neurons (MSNs) expressing either D1- or D2-type receptors [30]. D1- and D2-cells play an important role in drug dependence but their specific involvement in natural reward processing is far less known [31–33]. Therefore, we examined how natural and addictive reward treatment induces synaptic plasticity in D1 and D2 cells in the ACB. We focused on prolonged reward treatment as previous studies have shown that hallmarks of plastic changes in the ACB need a few days to manifest [33–35]. c-Fos expression pattern in D1 and D2 neurons is visualized in Fig. S6.

With a patch-clamp technique, we studied neuronal markers of synaptic plasticity—silent synapses. Such synapses are characterized by the imbalance of the two glutamatergic receptors: N-Methyl-D-aspartic acid receptors (NMDARs) and α-amino-3-hydroxy-5-methyl-4-isoxazolepropionic acid receptors (AMPARs). More precisely, silent synapses contain NMDARs but lack AMPARs, which makes them inactive at the resting state (hence the term “silent”). However, their generation in the adult brain indicates synaptic reorganization - either their formation or pruning [23, 36]. The generation of silent synapses in the ACB and their subsequent maturation into fully functional contacts has been strongly linked to the development of cocaine addiction-related behaviors such as drug seeking and drug craving [37, 38], cocaine-induced locomotor sensitization [39, 40], and cocaine-associated place preference [41].

**Fig. 5:**
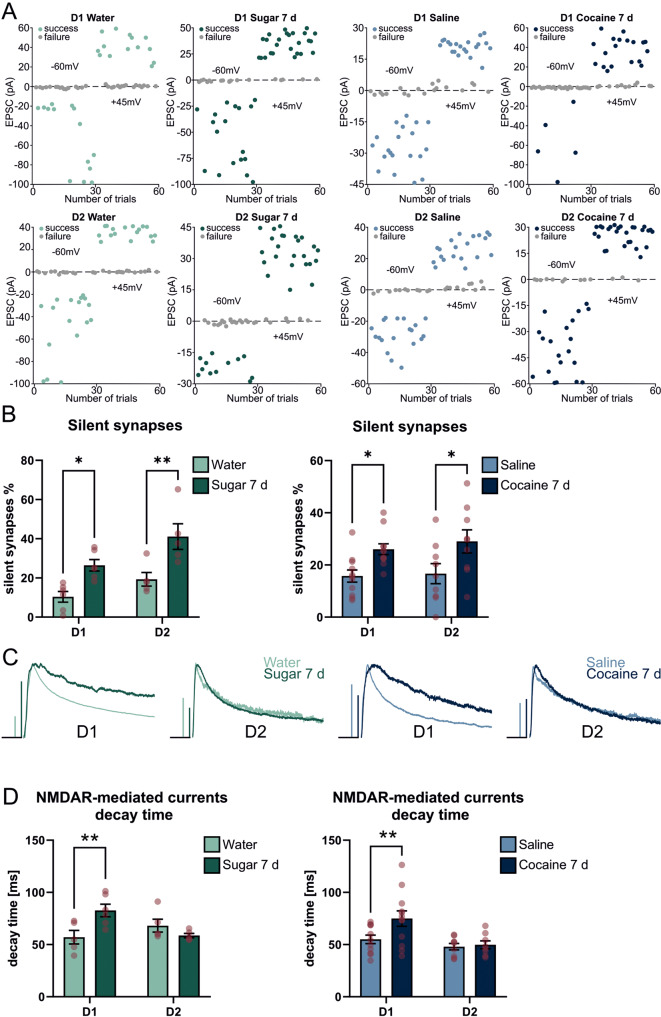
Electrophysiological determination of plastic changes of D1- and D2-positive cells in Nucleus Accumbens. **A** Exemplary recordings of AMPAR and NMDAR-mediated EPSCs from D1- (upper) and D2-positive (lower) cells from mice exposed to water (light green), sucrose (dark green), saline (light blue), and cocaine (dark blue). Each dot on the graph represents the amplitude of a single EPSC at resting membrane potential -60 mV (for AMPAR-mediated currents) and +45 mV (for NMDAR-mediated currents). Note almost ~50% success and ~50% failures in control groups at both -60 mV and + 40 mV. This ratio was disrupted in reward-exposed groups and more successes were observed at +45 mV for equal strength of electrical stimulation. **B** Percentage of silent synapses after natural (green) and pharmacological (blue) treatment. **C** Exemplary traces of NMDAR-mediated currents recorded from D1- (upper) and D2-positive (lower) cells from mice exposed to water (light green), sucrose (dark green), saline (light blue), and cocaine (dark blue). Horizontal bars are 25 ms. Vertical bars are 100 pA for D1: Water, Sugar 7d, Saline and 25 pA for D1 Cocaine 7d and D2 Water, Sugar 7 d, Saline, Cocaine 7 d. **D** Decay time of NMDAR-mediated EPSCs after natural (green) and pharmacological (blue) reward treatment. For silent synapses in D1-positive cells N Water = 6(16); N Sugar 7 d = 6(24); N Saline = 11(28), N Cocaine 7 d = 11(30) and for D2-positive cells N Water = 5(21); N Sugar 7 d = 5(26); N Saline = 9(28), N Cocaine 7d = 9(22), where N/n = Number of mice/ number of recorded neurons. For decay time in D1-positive cells N Water = 5(9); N Sugar 7 d = 6(11); N Saline = 11(25), N Cocaine 7 d = 12(21) and for D2-positive cells N Water = 5(20); N Sugar 7 d = 5(26); N Saline = 9(30), N Cocaine 7 d = 8(22), where N = Number of mice(Number of neurons). *p*-values were determined with the Two-way ANOVA test. *p < 0.05; **p < 0.01. For silent synapses *p*-value D1 Water vs. D1 Sugar 7 d = 0.0158; D2 Water vs. D2 Sugar 7 d = 0.0033; D1 Saline vs. D1 Cocaine 7 d = 0.0307; D2 Saline vs. D2 Cocaine 7 d = 0.0088. For decay time *p*-value D1 Water vs. D1 Sugar 7 d = 0.0084; D2 Water vs. D2 Sugar 7 d = 0.4475, D1 Saline vs. D1 Cocaine 7 d = 0.0056, D2 Saline vs. D2 Cocaine 7 d = 0.9701.

Our results show that both sucrose and cocaine exposure increases the number of silent synapses in the D1 and D2 cells of the ACB shell (Fig. 5A, B). The percentage of silent synapses in control groups was below 20%, which was exhibited by an almost equal number of successes and failures in response to the electrical stimulus. Exposure to both sugar and cocaine doubled the baseline level of silent synapses indicating an ongoing reorganization of the inputs to the ACB. Following the observation that cocaine-induced silent synapses in the ACB are new synaptic contacts, expressing GluN2B-containing NMDA receptors [23, 35, 39] we analyzed the decay kinetics of the NMDARs-mediated EPSCs in both rewards to verify the insertion of GluN2B subunits. These GluN2B-NMDAR variants are characterized by slower decay kinetics; thus, the lengthening of the decay time would indicate the increase in synaptic, GluN2B-NMDARs. Similarly to the abovementioned reports, exposure to sweet water lengthened the NMDARs’ currents kinetics indicating the formation of new synaptic contacts in response to sweet water self-administration. Exposure to cocaine showed a similar result, however, it did not reach the significance level. Lengthening of the decay time was observed only in the D1-expressing cell population (Fig. 5C, D). Together, these results indicate the high similarity of the plastic changes in the ACB shell: both sugar self-administration and cocaine i.p. injections, trigger the formation of silent synapses that, after maturation will enhance glutamatergic drive onto D1 neurons.

## Discussion

The present study used single-cell whole-brain imaging, as well as cellular techniques to determine how neuronal networks process natural and pharmacological rewards. The major findings can be summarized as follows. Exposure to rewards results in a massive activation of brain structures throughout the whole brain, yet the pattern of such activation differs for both types of rewards tested. A single exposure to sugar causes a widespread activation that fades with repeated exposure. On the other hand, brain activation after cocaine potentiates with repeated injections. On the cellular level, in the ACB, repeated exposure to both cocaine and sugar triggered identical hallmarks of synaptic reorganization. However, on the network level, significant differences were observed in brain modularity, which increased after single sugar exposure and both acute and repeated cocaine injections.

### The whole-brain mapping of the neuronal activity after drugs exposure

Brain clearing combined with IEGs labeling and light-sheet imaging offers unbiased, single-cell resolution analysis of the task-dependent neuronal activity in rodents. This approach has already served to characterize c-Fos expression elicited by drugs: medications regulating appetite, antipsychotics (haloperidol), and psychedelics (ketamine and psilocybin) [16, 42, 43]. c-Fos was also mapped in the brains of mice withdrawn from alcohol, psychostimulants, or opioids [12, 13, 21, 11]. Finally, the whole-brain approach was used to map cocaine’s effect on the level of expression of other IEGs, Arc, and Npas4. In that study, mice received two i.p. injections of 15 mg/kg cocaine or a foot shock, and IEGs expression was labeled 3 h after the last reward exposure [44]. Our results did not indicate c-Fos expression in the lateral and medial habenula, which are considered to be associated with aversive learning and depression-like symptoms [44, 45].

Overall, the abovementioned studies of plasticity markers triggered by a specific psychostimulant experience, support the hypothesis that drugs are processed by a distributed brain network [34, 46]. Together, these data will provide the scientific community with a platform that will serve as the basis for future studies in greater detail. Such mapping of drug-activated structures, followed by a comprehensive analysis of neuronal adaptations on cellular and molecular levels, is significant for at least two reasons. Firstly, knowing the molecular targets of drugs of abuse may help to choose receptor-specific medications for dependence. Secondly, it is hypothesized that reversing the drug-related plastic changes of specific neuronal ensembles may erase the memories associated with addictions [34]. Furthermore, understanding whether drugs of abuse are processed by neuronal ensembles relevant for other rewards may help mitigate side effects of drug withdrawal, such as anhedonia.

### Cellular adaptations of the ACB after reward exposure

In our study, we investigated the reward-evoked plasticity in the ACB. We particularly focused on this structure, as the whole-brain analysis pointed to its activation by either reward exposure. As the anatomical structure of the ACB is well-described, with two, non-overlapping dopamine-sensitive cell populations: D1 and D2 cells, we specifically focused on plasticity markers in these two distinct populations, in response to natural and addictive rewards. Notably, dopamine has opposite effects on these two cellular populations, as D1- and D2-type receptors are coupled with G-proteins that, respectively, stimulate and inhibit the synthesis of cAMP, thus triggering different signaling cascades and therefore associating these neurons with different functions [47].

Previous studies have shown that the ACB is a crucial neuronal substrate through which cocaine experience induces persistent synaptic and circuit adaptations to promote drug-seeking [33]. Moreover, cocaine has been shown to engage both D1 and D2 cells but during different steps of dependence [31, 32]. There seems to be a consensus about the strengthening of the glutamatergic drive onto D1 MSNs by cocaine exposure, as electrophysiological and imaging studies revealed increased AMPARs transmission, insertion of calcium-permeable AMPARs, and de novo formation of new synapses [23, 48–51]. Moreover, studies suggested the weakening of excitatory synapses on D2 cells after the drug treatment [48, 49] or no modifications of D2 cells 1 day or 7–10 days after the 5-day cocaine exposure [23, 51]. The involvement of the ACB in natural reward-related learning is less known, but it was shown that a high-fat diet triggers silent synapses in the ACB core, followed by insertion of calcium-permeable AMPARs, increases AMPAR/NMDA currents ratio and dendritic spine density, suggesting overall enhancement of glutamatergic drive onto MSNs in the ACB core [52–54]. Also, the increase of c-Fos levels in D1 and D2 cells in the ACB was observed during sucrose-seeking [31].

In our study, using two different reward exposure protocols, we were able to directly show that both cocaine and sucrose treatments triggered identical cellular changes in glutamatergic transmission. Cocaine and sucrose similarly strengthen excitatory synapses on D1 and, presumably, weaken those on D2 neurons. Taken together, molecular changes induced by natural and pharmacological reward exposure share a lot of common points in the ACB, which further supports the hypothesis that natural and maladaptive forms of learning tap into the same mechanisms. Our results could also suggest an addictive aspect of natural rewards, as excitatory transmission from the mPFC to the ACB is involved in compulsive food-seeking [55].

### Brain structures activated by sugar and cocaine exposures

With such a massive brain activation triggered by two different rewards, we observed c-Fos expression in all major brain systems. We measured a strong activation of the hippocampal formation, including the subiculum and entorhinal cortex, suggestive of the impact of a new context, spatial and non-spatial features of the place where the rewards were administered [56]. Activation of the amygdalar complex, responsible for negative (aversive) associations, such as the lateral and anterior basolateral amygdala projections to the central nuclei [57] could represent the surprise and stress of the first exposure to sugar, as well as fear of the i.p. injection in the case of cocaine. At the same time, c-Fos expression in the parts of the amygdala that process positive reinforcement, such as the posterior basolateral nucleus and the central amygdala would represent the rewarding aspects of sugar and cocaine exposures [57–59]. Together the result shows the ongoing computation and valence assignment to the rewards experiences. The robust global brain activation triggered by the first sugar exposure does not appear after repeated exposure, which suggests that the novelty of the reward is no longer experienced. Similarly, in a recent study on palatable food seeking, the researchers observed that 7 days of high-carbohydrate diet consumption, didn’t significantly induce c-Fos expression compared to control, home cage animals. However, nearly all major brain subdivisions, such as the cortical subplate, isocortex, olfactory areas, hippocampus, hindbrain, and striatum were activated after 60 days of abstinence, indicating incubation of food craving [10].

It could appear surprising, that cocaine i.p. injections did not significantly induce c-Fos expression in the VTA. Studies have shown that in rats which preferred saccharin over cocaine (both self-administered) a single exposure to the sweet taste reduced, while cocaine increased c-Fos expression in the VTA [14]. Moreover, a single cocaine i.p. injection potentiates glutamatergic transmission in the VTA in a form of an increased AMPA/NMDA ratio lasting between 5–10 days [60, 61]. Our results showed an increase in the number of c-Fos positive neurons, but only exposure to sweet water (single and repeated) exceeded the significance threshold. An explanation could be inferred from another study, comparing LTP induction by repeated cocaine, food, and sucrose self-administration, and cocaine passive i.v. injections [62]. Only self-administered cocaine induced a long-lasting increase in the strength of glutamatergic synapses in the VTA. Food and sugar induced only a transient potentiation [62]. The explanation could be that a strong, persistent plasticity in the VTA is induced when an association is formed between the task and the reward. In our paradigm, passive i.p. injections, which induce locomotor sensitization were not a suitable model of reinforced learning that would trigger strong plasticity in the VTA. Our imaging result confirmed that.

Another interesting observation made with a holistic look at the whole brain was a strong activation of the visual and auditory cortex. The effect was present for both rewards but was slightly stronger after cocaine exposure. It was reported that cocaine injection triggers dopamine release in the auditory and visual cortex in a dose-dependent manner [63]. Others reported an increase in glutamate turnover in the visual cortex in cocaine self-administering rats [64]. What is more, the activity in the visual cortex is highly modulated by the behavioral state, arousal [65], and locomotion [66]. Running increases the activity of the primary visual cortex (V1) layer 2/3 neurons in adult mice [67]. Both rewards used in our study induced the c-Fos levels in the V1 layer 2/3. Visual stimuli incoming from the retina are processed via two distinct pathways – through the thalamus into the visual cortex and through the superior colliculus (SC) [68]. The SC is a structure that enables sensory-to-motor transformations. This includes incorporating the vision, audition, and somatosensation to issue motor commands [69]. Single and repeated sugar exposure activated the SC layers responsible for visual, sensory, and motor functions. As expected, it shows that obtaining the sweet reward requires processing a variety of stimuli that will allow the mouse to choose and approach the sweet water bottle. In the case of passive cocaine i.p. injection, the activation of the SC appears only after repeated exposure, thus after the expression of the locomotor sensitization. None of the midbrain structures expressed c-Fos after a single cocaine injection. This further highlights the differences between self-administered sugar vs. passively injected pharmacological reward.

### The global activation of the brain after reward exposure

Whole-brain c-Fos mapping unraveled that both sweet water and cocaine exposure produced a widespread activation of distant neuronal networks. Likewise, in other studies, the whole-brain mapping of the IEGs expression level after the aversive learning has shown a large-fold increase of the c-Fos level throughout almost the entire brain [7]. The wide neuronal circuitry (117/247 brain regions) is involved in the fear response. These findings from c-Fos labeling are consistent with other experiments, proving the activation of a significant portion of the animal brain during complex behaviors [70–72]. Correspondingly, studies on humans have shown the contribution of the extensive neural network in some cognitive tasks [73]. Together, all these findings support the hypothesis of brain-wide task-related activity in rodents and humans [74, 75]. These results stress the importance of whole-brain studies for the understanding of neuronal computation.

We used hierarchical clustering of all studied brain structures to identify the global pattern of neuronal reorganization after reward exposure. c-Fos signal level worked to organize brain regions into modules, which are thought to represent groups of neuronal networks that are collectively involved in a response to the task [76, 77]. In principle, increased, hierarchical brain modularity i.e. brain structures organized into many, functionally connected modules, facilitates adaptation to the changing surrounding world. When one or several modules adapt to the novel stimulus, the entire network benefits from integrating this new change, while preserving the overall network function with other, stable, higher-level modules [78]. Thus, increased brain modularity has been proposed as a biomarker of plasticity and was found in humans performing cognitive training [28, 79]. However, such network reorganization was also a result of disease (such as schizophrenia), or exposure to drugs of abuse and was linked to addiction [80–83]. In animals, both alcohol and cocaine withdrawal caused a decline in the modularity of the brain, indicating that abstinence is associated with a reduction in the brain’s functional connectivity [13, 21]. Contradictory, we found that cocaine-exposed mice exhibited a higher number of small modules of co-activated brain regions. We also observed brain connectivity alterations after single, but not prolonged treatment with sucrose. Therefore, we hypothesize that changes in the brain modularity caused by sugar exposure were a robust, transient response to the novelty. In a comparative study, rats presented with saccharin and intravenous cocaine almost exclusively chose the sweet taste over the addictive drug [84]. Therefore, experiencing sweetness exerts a strong brain activation representing the immense salience of the first exposure to sugar. Repeated sugar intake does not enhance further this initial activation. On the other hand, drugs with their pharmacological effects are described as stimuli, which valence does not decrease over repeated use [85]. They cause long-lasting alterations in the functional connectivity of brain regions modulated by dopamine, which are linked to the decreased reward sensitivity reported in cocaine abusers [86]. Thus, the salience of cocaine reward develops with repeated use, which ultimately leads to a compulsion.

The brain pattern of c-Fos activation changes over the animal task performance time, e.g., different brain ensembles are activated at different steps of fear-based learning [9, 87]. Also, it was shown that acute and repeated self-administration of cocaine may involve different neural ensembles [88]. Finally, in some brain regions, the elevated c-Fos level is highly correlated with the novelty of the stimulus [89–91]. Here, we show that repeated cocaine exposure is associated with more activated structures than acute exposure. It might represent the pharmacological property of cocaine to constantly elevate dopamine release, signaling that the reward is always better than expected [85]. Alternatively, since passive i.p. injections are not as potent as self-administration and the reward is not instant (many seconds after the i.p. injection) the increase in the number of activated brain regions after repeated exposure could represent the association formed between the i.p. injection, the context of the locomotion measurements setup and the pleasure of cocaine’s pharmacological effect. Exposure to sucrose, on the other hand, displayed the opposite pattern, where single but not prolonged exposure caused more robust effects.

### Limitations of the study

Cocaine and sucrose clearly differ in their pharmacological effects on cells, but also concerning their smell and palatability. Therefore, our studies were limited by the route of administration of these substances. We are aware that some of the observed changes in the brain may be the results of the administration route, rather than reward processing itself. Still, we decided to use intraperitoneal cocaine injections, as such an approach is validated in many laboratories, offers precise control over drug concentration, and is replicable in a group of animals. We have considered two other models: self-administration or consumption of cocaine solution. In the first scenario, a mouse undergoes a complicated surgery. After recovery, a mouse has to be pre-trained to lever-press or nose-poke to obtain the reward. Moreover, operant action is often strengthened with sound or visual cues. Such various stimuli would trigger plasticity markers in brain regions unrelated to the reward itself and would complicate the interpretation of the results from a single cocaine exposure. However, imaging the brain-wide plasticity pattern of long-term cocaine self-administration, combined with periods of withdrawal and incubation of drug craving would bring highly valuable information on how addiction development is reflected in complex brain networks. In the second model, a mouse has to be progressively exposed to increasing concentrations of cocaine diluted in drinking water. Moreover, due to cocaine’s bitterness, a sweetener has to be used to minimize aversion and increase the motivation for drinking, making it impossible to dissociate the effects of cocaine alone.

As an indirect marker of the pattern of neuronal activity, we used c-Fos immunoreactivity. The *c-fos* gene belongs to a group of IEGs activated upon a high stimulation associated with learning but also with the development of addiction [92]. The limitation of using c-Fos for studying brain activity is the unidimensionality of this method, as protein level is measured only at one specific time point. However, it has also a unique benefit, as IEGs are thought to mediate critical steps in protein synthesis, synaptic potentiation, and structural plasticity. Thus, c-Fos is used not only as a marker of neuronal activity but also the structural plasticity [26, 93].

## Supplementary information

Supplementary Material
