# Supplementary material for: Whole-brain tracking of cocaine and sugar rewards processing
Source: Transl Psychiatry. 2023 Jan 23;13:20. doi: 10.1038/s41398-023-02318-4 (PMC9868126; doi:10.1038/s41398-023-02318-4)
Supplement: Supplementary file 1 — Supplementary Material [file 41398_2023_2318_MOESM1_ESM.pdf]

Supplementary Information

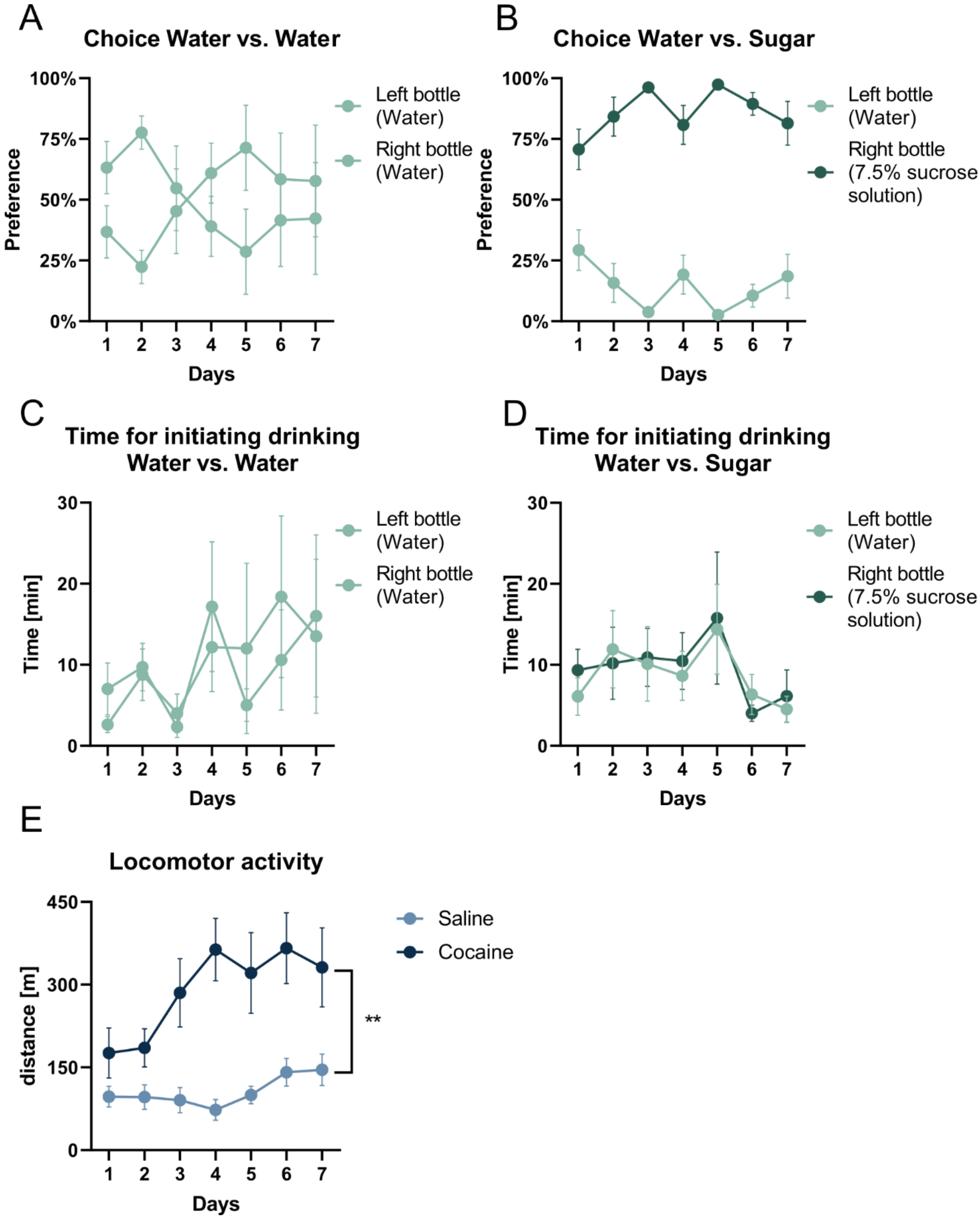

Fig. Supp. 1: **Mice responses to natural (green) and addictive (blue) rewards.**

A) Percentage of mice preference of drinking from two separate bottles filled with water. **B)** Percentage of mice preference of drinking from two separate bottles filled with water (shallow green) or 7.5 % sucrose solution (dark green). **C, D)** The latency to the first lick from bottles filled with water (light green) or 7.5 % sucrose solution (dark green). Panel **C** shows the control group with two water bottles, while panel **D** shows the reward-treated group with access to normal and sweet water. **E)** Locomotor activity of mice treated with either saline or cocaine IP injections. N Water=9, N Sugar 7 d=9, N Saline=11, N Cocaine 7 d=13.**=p<0.001.

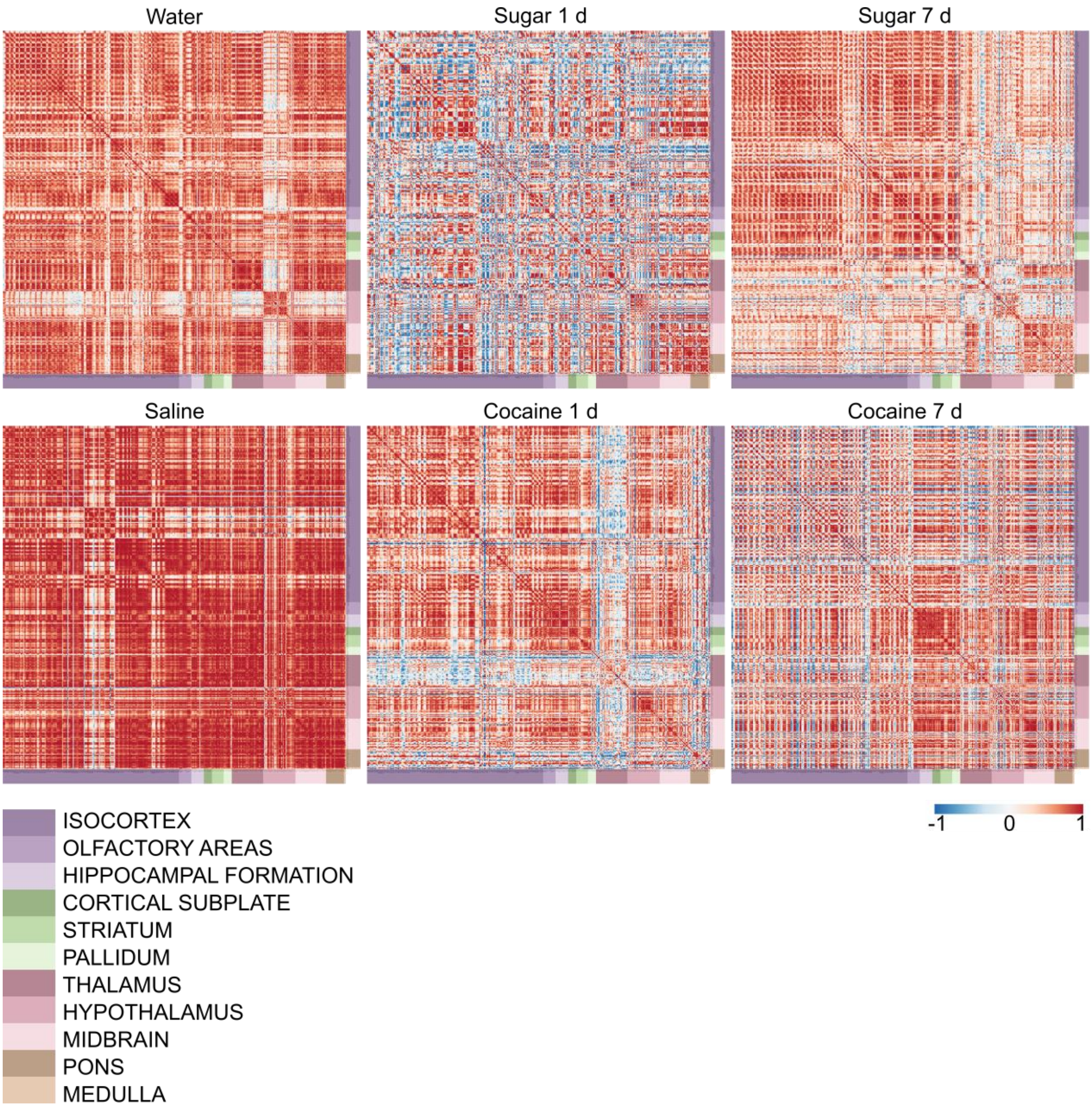

Fig. Supp. 2: **Brain region correlation heatmaps organized according to the Allen Brain Atlas.** Correlation map for groups treated with natural (upper row) and pharmacological (lower row) rewards. On each heatmap's left and right edges are abbreviations of the brain structures. They were grouped into the anatomical organization, which was marked with colors. Color-coded anatomical annotations are depicted below the heatmaps. On heatmaps, the correlation strength between single structures is marked with warmer colors indicating more similar signal densities between two structures in all mice. N Water=7, N Sugar 1 d=5, N Sugar 7 d=7, N Saline=7, N Cocaine 1 d=6, N Cocaine 7 d=6.

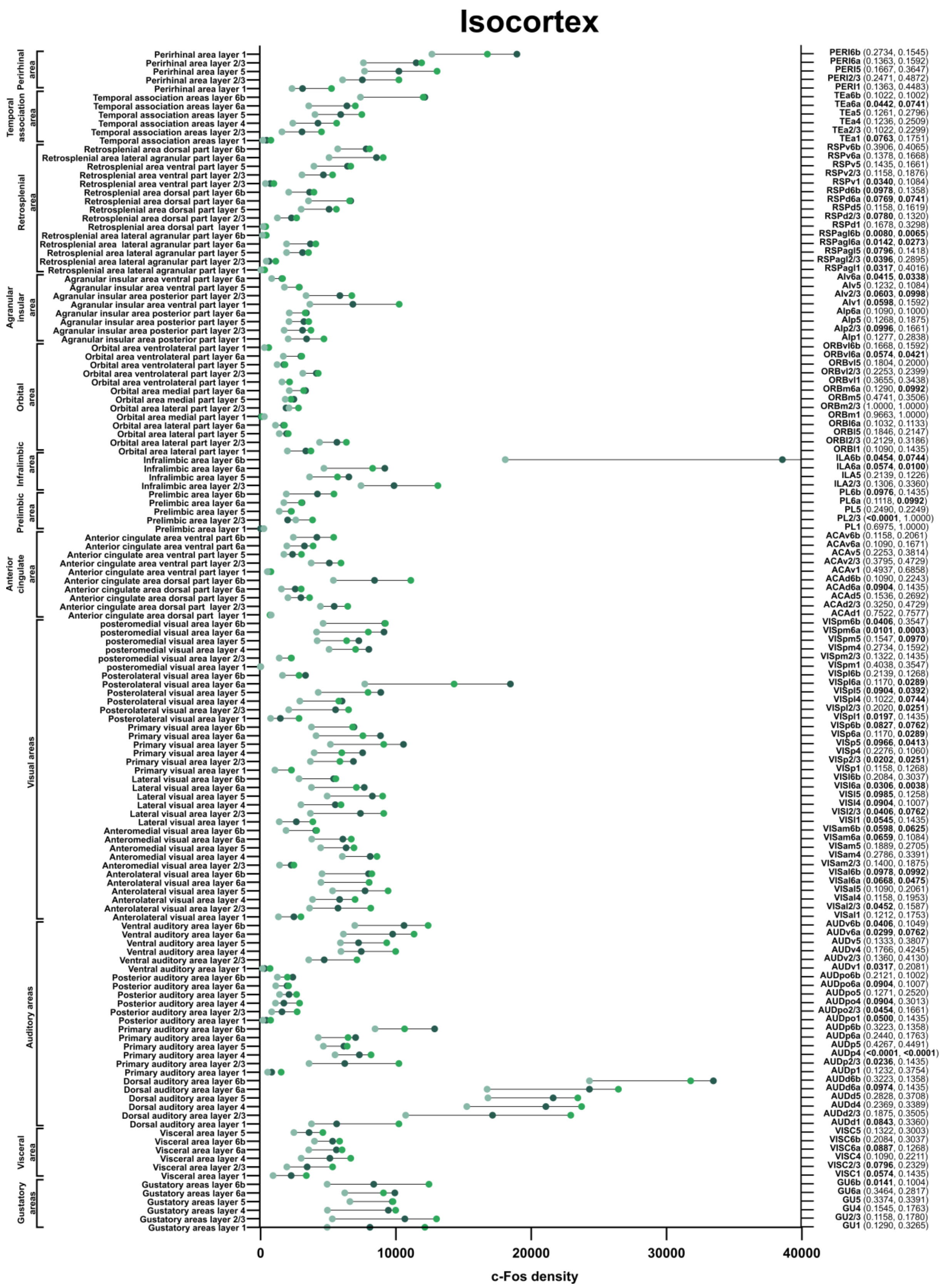
Isocortex
Perirhinal area
Temporal association area
Retrosplenial area
Agranular insular area
Orbital area
Infralimbic area
Prelimbic area
Anterior cingulate area
Visual areas
Auditory areas
Visceral area
Gustatory areas
Perirhinal area layer 1
Perirhinal area layer 2/3
Perirhinal area layer 5
Perirhinal area layer 2/3
Perirhinal area layer 1
Temporal association areas layer 6b
Temporal association areas layer 6a
Temporal association areas layer 5
Temporal association areas layer 4
Temporal association areas layer 2/3
Temporal association areas layer 1
Retrosplenial area dorsal part layer 6b
Retrosplenial area lateral agranular part layer 6a
Retrosplenial area ventral part layer 5
Retrosplenial area ventral part layer 2/3
Retrosplenial area ventral part layer 2/3
Retrosplenial area dorsal part layer 6b
Retrosplenial area dorsal part layer 6a
Retrosplenial area dorsal part layer 5
Retrosplenial area dorsal part layer 2/3
Retrosplenial area dorsal part layer 1
Retrosplenial area lateral agranular part layer 6b
Retrosplenial area lateral agranular part layer 6a
Retrosplenial area lateral agranular part layer 5
Retrosplenial area lateral agranular part layer 2/3
Retrosplenial area lateral agranular part layer 1
Agranular insular area ventral part layer 6a
Agranular insular area ventral part layer 5
Agranular insular area posterior part layer 2/3
Agranular insular area ventral part layer 1
Agranular insular area posterior part layer 6a
Agranular insular area posterior part layer 5
Agranular insular area posterior part layer 2/3
Agranular insular area posterior part layer 1
Orbital area ventrolateral part layer 1
Orbital area ventrolateral part layer 6a
Orbital area ventrolateral part layer 5
Orbital area ventrolateral part layer 2/3
Orbital area ventrolateral part layer 1
Orbital area medial part layer 6a
Orbital area medial part layer 5
Orbital area lateral part layer 2/3
Orbital area medial part layer 1
Orbital area lateral part layer 6a
Orbital area lateral part layer 5
Orbital area lateral part layer 2/3
Orbital area lateral part layer 1
Infralimbic area layer 6b
Infralimbic area layer 6a
Infralimbic area layer 5
Infralimbic area layer 2/3
Prelimbic area layer 6b
Prelimbic area layer 6a
Prelimbic area layer 5
Prelimbic area layer 2/3
Prelimbic area layer 1
Anterior cingulate area ventral part 6b
Anterior cingulate area ventral part 6a
Anterior cingulate area ventral part layer 5
Anterior cingulate area ventral part layer 2/3
Anterior cingulate area ventral part layer 1
Anterior cingulate area dorsal part layer 6b
Anterior cingulate area dorsal part layer 6a
Anterior cingulate area dorsal part layer 5
Anterior cingulate area dorsal part layer 2/3
Anterior cingulate area dorsal part layer 1
posteromedial visual area layer 6b
posteromedial visual area layer 6a
posteromedial visual area layer 5
posteromedial visual area layer 4
posteromedial visual area layer 2/3
posteromedial visual area layer 1
Posterolateral visual area layer 6b
Posterolateral visual area layer 6a
Posterolateral visual area layer 5
Posterolateral visual area layer 4
Posterolateral visual area layer 2/3
Posterolateral visual area layer 1
Primary visual area layer 6b
Primary visual area layer 6a
Primary visual area layer 5
Primary visual area layer 4
Primary visual area layer 2/3
Primary visual area layer 1
Lateral visual area layer 6b
Lateral visual area layer 6a
Lateral visual area layer 5
Lateral visual area layer 4
Lateral visual area layer 2/3
Lateral visual area layer 1
Anteromedial visual area layer 6b
Anteromedial visual area layer 6a
Anteromedial visual area layer 5
Anteromedial visual area layer 4
Anteromedial visual area layer 2/3
Anterolateral visual area layer 6b
Anterolateral visual area layer 6a
Anterolateral visual area layer 5
Anterolateral visual area layer 4
Anterolateral visual area layer 2/3
Anterolateral visual area layer 1
Ventral auditory area layer 6b
Ventral auditory area layer 6a
Ventral auditory area layer 5
Ventral auditory area layer 4
Ventral auditory area layer 2/3
Ventral auditory area layer 1
Posterior auditory area layer 6b
Posterior auditory area layer 6a
Posterior auditory area layer 5
Posterior auditory area layer 4
Posterior auditory area layer 2/3
Posterior auditory area layer 1
Primary auditory area layer 6b
Primary auditory area layer 6a
Primary auditory area layer 5
Primary auditory area layer 4
Primary auditory area layer 2/3
Primary auditory area layer 1
Dorsal auditory area layer 6b
Dorsal auditory area layer 6a
Dorsal auditory area layer 5
Dorsal auditory area layer 4
Dorsal auditory area layer 2/3
Dorsal auditory area layer 1
Visceral area layer 5
Visceral area layer 6b
Visceral area layer 6a
Visceral area layer 4
Visceral area layer 2/3
Visceral area layer 1
Gustatory areas layer 6b
Gustatory areas layer 6a
Gustatory areas layer 5
Gustatory areas layer 4
Gustatory areas layer 2/3
Gustatory areas layer 1
0
10000
20000
30000
40000
c-Fos density
PERI6b (0.2734, 0.1545)
PERI6a (0.1363, 0.1592)
PERI5 (0.1667, 0.3647)
PERI2/3 (0.2471, 0.4872)
PERI1 (0.1363, 0.4483)
TEa6b (0.1022, 0.1002)
TEa6a (0.0442, 0.0741)
TEa5 (0.1261, 0.2796)
TEa4 (0.1236, 0.2509)
TEa2/3 (0.1022, 0.2299)
TEa1 (0.0763, 0.1751)
RSPv6b (0.3906, 0.4065)
RSPv6a (0.1378, 0.1668)
RSPv5 (0.1435, 0.1661)
RSPv2/3 (0.1158, 0.1876)
RSPv1 (0.0340, 0.1084)
RSPd6b (0.0978, 0.1358)
RSPd6a (0.0769, 0.0741)
RSPd5 (0.1158, 0.1619)
RSPd2/3 (0.0780, 0.1320)
RSPd1 (0.1678, 0.3298)
RSPagl6b (0.0080, 0.0065)
RSPagl6a (0.0142, 0.0273)
RSPagl5 (0.0796, 0.1418)
RSPagl2/3 (0.0396, 0.2895)
RSPagl1 (0.0317, 0.4016)
AIv6a (0.0415, 0.0338)
AIv5 (0.1232, 0.1084)
AIv2/3 (0.0603, 0.0998)
AIv1 (0.0598, 0.1592)
AIp6a (0.1090, 0.1000)
AIp5 (0.1268, 0.1875)
AIp2/3 (0.0996, 0.1661)
AIp1 (0.1277, 0.2838)
ORBvl6b (0.1668, 0.1592)
ORBvl6a (0.0574, 0.0421)
ORBvl5 (0.1804, 0.2000)
ORBvl2/3 (0.2253, 0.2399)
ORBvl1 (0.3655, 0.3438)
ORBm6a (0.1290, 0.0992)
ORBm5 (0.4741, 0.3506)
ORBm2/3 (1.0000, 1.0000)
ORBm1 (0.9663, 1.0000)
ORBl6a (0.1032, 0.1133)
ORBl5 (0.1846, 0.2147)
ORBl2/3 (0.2129, 0.3186)
ORBl1 (0.1090, 0.1435)
ILA6b (0.0454, 0.0744)
ILA6a (0.0574, 0.0100)
ILA5 (0.2139, 0.1226)
ILA2/3 (0.1306, 0.3360)
PL6b (0.0976, 0.1435)
PL6a (0.1118, 0.0992)
PL5 (0.2490, 0.2249)
PL2/3 (<0.0001, 1.0000)
PL1 (0.6975, 1.0000)
ACAv6b (0.1158, 0.2061)
ACAv6a (0.1090, 0.1671)
ACAv5 (0.2253, 0.3814)
ACAv2/3 (0.3795, 0.4729)
ACAv1 (0.4937, 0.6858)
ACAd6b (0.1090, 0.2243)
ACAd6a (0.0904, 0.1435)
ACAd5 (0.1536, 0.2692)
ACAd2/3 (0.3250, 0.4729)
ACAd1 (0.7522, 0.7577)
VISpm6b (0.0406, 0.3547)
VISpm6a (0.0101, 0.0003)
VISpm5 (0.1547, 0.0970)
VISpm4 (0.2734, 0.1592)
VISpm2/3 (0.1322, 0.1435)
VISpm1 (0.4038, 0.3547)
VISpl6b (0.2139, 0.1268)
VISpl6a (0.1170, 0.0289)
VISpl5 (0.0904, 0.0392)
VISpl4 (0.1022, 0.0744)
VISpl2/3 (0.2020, 0.0251)
VISpl1 (0.0197, 0.1435)
VISp6b (0.0827, 0.0762)
VISp6a (0.1170, 0.0289)
VISp5 (0.0966, 0.0413)
VISp4 (0.2276, 0.1060)
VISp2/3 (0.0202, 0.0251)
VISp1 (0.1158, 0.1268)
VISl6b (0.2084, 0.3037)
VISl6a (0.0306, 0.0038)
VISl5 (0.0985, 0.1258)
VISl4 (0.0904, 0.1007)
VISl2/3 (0.0406, 0.0762)
VISl1 (0.0545, 0.1435)
VISam6b (0.0598, 0.0625)
VISam6a (0.0659, 0.1084)
VISam5 (0.1889, 0.2705)
VISam4 (0.2786, 0.3391)
VISam2/3 (0.1400, 0.1875)
VISal6b (0.0978, 0.0992)
VISal6a (0.0668, 0.0475)
VISal5 (0.1090, 0.2061)
VISal4 (0.1158, 0.1953)
VISal2/3 (0.0452, 0.1587)
VISal1 (0.1212, 0.1753)
AUDv6b (0.0406, 0.1049)
AUDv6a (0.0299, 0.0762)
AUDv5 (0.1333, 0.3807)
AUDv4 (0.1766, 0.4245)
AUDv2/3 (0.1360, 0.4130)
AUDv1 (0.0317, 0.2081)
AUDpo6b (0.2121, 0.1002)
AUDpo6a (0.0904, 0.1007)
AUDpo5 (0.1271, 0.2520)
AUDpo4 (0.0904, 0.3013)
AUDpo2/3 (0.0454, 0.1661)
AUDpo1 (0.0500, 0.1435)
AUDp6b (0.3223, 0.1358)
AUDp6a (0.2440, 0.1763)
AUDp5 (0.4267, 0.4491)
AUDp4 (<0.0001, <0.0001)
AUDp2/3 (0.0236, 0.1435)
AUDp1 (0.1232, 0.3754)
AUDd6b (0.3223, 0.1358)
AUDd6a (0.0974, 0.1435)
AUDd5 (0.2828, 0.3708)
AUDd4 (0.2369, 0.3389)
AUDd2/3 (0.1875, 0.3505)
AUDd1 (0.0843, 0.3360)
VISC5 (0.1322, 0.3003)
VISC6b (0.2084, 0.3037)
VISC6a (0.0887, 0.1268)
VISC4 (0.1090, 0.2211)
VISC2/3 (0.0796, 0.2329)
VISC1 (0.0574, 0.1435)
GU6b (0.0141, 0.1004)
GU6a (0.3464, 0.2817)
GU5 (0.3374, 0.3391)
GU4 (0.1545, 0.1763)
GU2/3 (0.1158, 0.1780)
GU1 (0.1290, 0.3265)

Isocortex continued

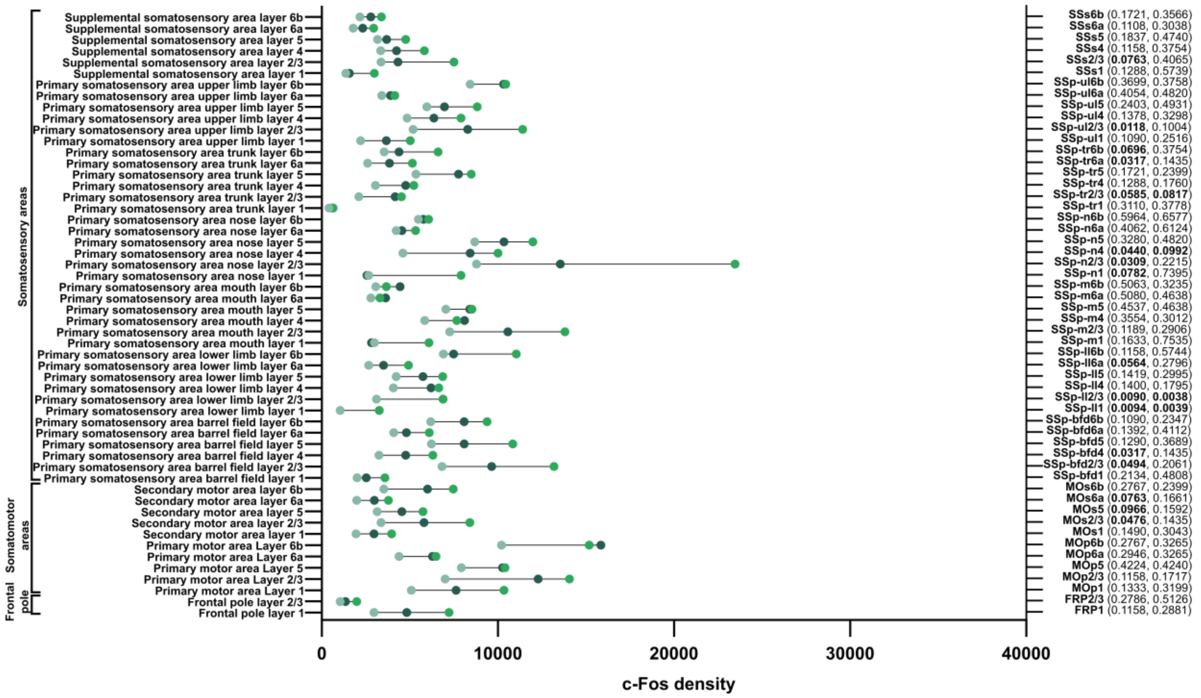

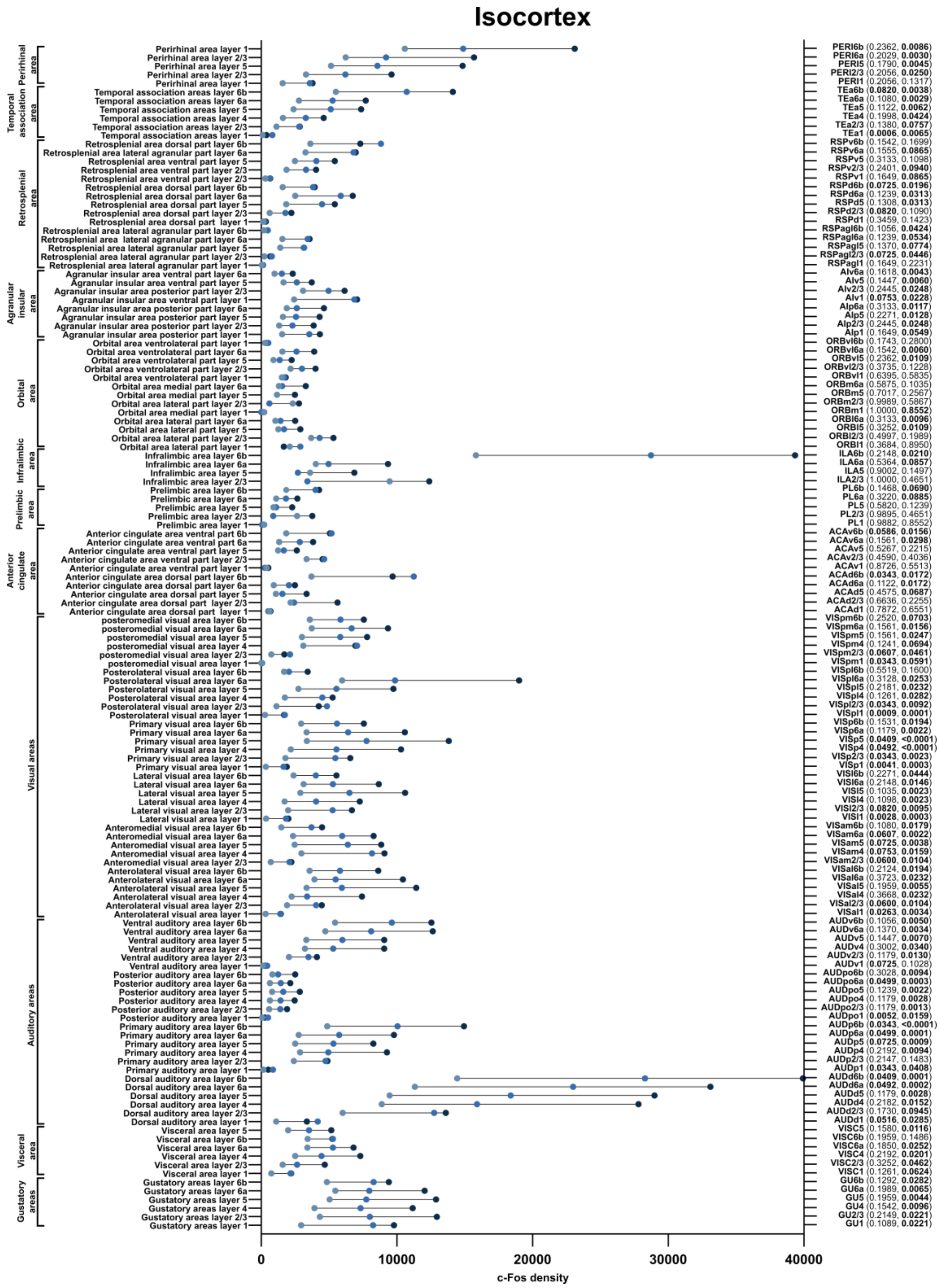
Isocortex
Perirhinal area
Temporal association area
Retrosplenial area
Agranular insular area
Orbital area
Infralimbic area
Prelimbic area
Anterior cingulate area
Visual areas
Auditory areas
Visceral area
Gustatory areas
Perirhinal area layer 1
Perirhinal area layer 2/3
Perirhinal area layer 5
Perirhinal area layer 2/3
Perirhinal area layer 1
Temporal association areas layer 6b
Temporal association areas layer 6a
Temporal association areas layer 5
Temporal association areas layer 4
Temporal association areas layer 2/3
Temporal association areas layer 1
Retrosplenial area dorsal part layer 6b
Retrosplenial area lateral agranular part layer 6a
Retrosplenial area ventral part layer 5
Retrosplenial area ventral part layer 2/3
Retrosplenial area ventral part layer 2/3
Retrosplenial area dorsal part layer 6b
Retrosplenial area dorsal part layer 6a
Retrosplenial area dorsal part layer 5
Retrosplenial area dorsal part layer 2/3
Retrosplenial area dorsal part layer 1
Retrosplenial area lateral agranular part layer 6b
Retrosplenial area lateral agranular part layer 6a
Retrosplenial area lateral agranular part layer 5
Retrosplenial area lateral agranular part layer 2/3
Retrosplenial area lateral agranular part layer 1
Agranular insular area ventral part layer 6a
Agranular insular area ventral part layer 5
Agranular insular area posterior part layer 2/3
Agranular insular area ventral part layer 1
Agranular insular area posterior part layer 6a
Agranular insular area posterior part layer 5
Agranular insular area posterior part layer 2/3
Agranular insular area posterior part layer 1
Orbital area ventrolateral part layer 1
Orbital area ventrolateral part layer 6a
Orbital area ventrolateral part layer 5
Orbital area ventrolateral part layer 2/3
Orbital area ventrolateral part layer 1
Orbital area medial part layer 6a
Orbital area medial part layer 5
Orbital area lateral part layer 2/3
Orbital area medial part layer 1
Orbital area lateral part layer 6a
Orbital area lateral part layer 5
Orbital area lateral part layer 2/3
Orbital area lateral part layer 1
Infralimbic area layer 6b
Infralimbic area layer 6a
Infralimbic area layer 5
Infralimbic area layer 2/3
Prelimbic area layer 6b
Prelimbic area layer 6a
Prelimbic area layer 5
Prelimbic area layer 2/3
Prelimbic area layer 1
Anterior cingulate area ventral part layer 6b
Anterior cingulate area ventral part layer 6a
Anterior cingulate area ventral part layer 5
Anterior cingulate area ventral part layer 2/3
Anterior cingulate area ventral part layer 1
Anterior cingulate area dorsal part layer 6b
Anterior cingulate area dorsal part layer 6a
Anterior cingulate area dorsal part layer 5
Anterior cingulate area dorsal part layer 2/3
Anterior cingulate area dorsal part layer 1
posteromedial visual area layer 6b
posteromedial visual area layer 6a
posteromedial visual area layer 5
posteromedial visual area layer 4
posteromedial visual area layer 2/3
posteromedial visual area layer 1
Posterolateral visual area layer 6b
Posterolateral visual area layer 6a
Posterolateral visual area layer 5
Posterolateral visual area layer 4
Posterolateral visual area layer 2/3
Posterolateral visual area layer 1
Primary visual area layer 6b
Primary visual area layer 6a
Primary visual area layer 5
Primary visual area layer 4
Primary visual area layer 2/3
Primary visual area layer 1
Lateral visual area layer 6b
Lateral visual area layer 6a
Lateral visual area layer 5
Lateral visual area layer 4
Lateral visual area layer 2/3
Lateral visual area layer 1
Anteromedial visual area layer 6b
Anteromedial visual area layer 6a
Anteromedial visual area layer 5
Anteromedial visual area layer 4
Anteromedial visual area layer 2/3
Anterolateral visual area layer 6b
Anterolateral visual area layer 6a
Anterolateral visual area layer 5
Anterolateral visual area layer 4
Anterolateral visual area layer 2/3
Anterolateral visual area layer 1
Ventral auditory area layer 6b
Ventral auditory area layer 6a
Ventral auditory area layer 5
Ventral auditory area layer 4
Ventral auditory area layer 2/3
Ventral auditory area layer 1
Posterior auditory area layer 6b
Posterior auditory area layer 6a
Posterior auditory area layer 5
Posterior auditory area layer 4
Posterior auditory area layer 2/3
Posterior auditory area layer 1
Primary auditory area layer 6b
Primary auditory area layer 6a
Primary auditory area layer 5
Primary auditory area layer 4
Primary auditory area layer 2/3
Primary auditory area layer 1
Dorsal auditory area layer 6b
Dorsal auditory area layer 6a
Dorsal auditory area layer 5
Dorsal auditory area layer 4
Dorsal auditory area layer 2/3
Dorsal auditory area layer 1
Visceral area layer 5
Visceral area layer 6b
Visceral area layer 6a
Visceral area layer 4
Visceral area layer 2/3
Visceral area layer 1
Gustatory areas layer 6b
Gustatory areas layer 6a
Gustatory areas layer 5
Gustatory areas layer 4
Gustatory areas layer 2/3
Gustatory areas layer 1
PERI6b (0.2362, 0.0086)
PERI6a (0.2029, 0.0030)
PERI5 (0.1790, 0.0045)
PERI2/3 (0.2056, 0.0250)
PERI1 (0.2056, 0.1317)
TEa6b (0.0820, 0.0038)
TEa6a (0.1080, 0.0029)
TEa5 (0.1122, 0.0062)
TEa4 (0.1998, 0.0424)
TEa2/3 (0.1380, 0.0757)
TEa1 (0.0006, 0.0065)
RSPv6b (0.1542, 0.1699)
RSPv6a (0.1555, 0.0865)
RSPv5 (0.3133, 0.1098)
RSPv2/3 (0.2401, 0.0940)
RSPv1 (0.1649, 0.0865)
RSPd6b (0.0725, 0.0196)
RSPd6a (0.1239, 0.0313)
RSPd5 (0.1308, 0.0313)
RSPd2/3 (0.0820, 0.1090)
RSPd1 (0.3459, 0.1423)
RSPagl6b (0.1056, 0.0424)
RSPagl6a (0.1239, 0.0534)
RSPagl5 (0.1370, 0.0774)
RSPagl2/3 (0.0725, 0.0446)
RSPagl1 (0.1649, 0.2231)
AIv6a (0.1618, 0.0043)
AIv5 (0.1447, 0.0060)
AIv2/3 (0.2445, 0.0248)
AIv1 (0.0753, 0.0228)
AIp6a (0.3133, 0.0117)
AIp5 (0.2271, 0.0128)
AIp2/3 (0.2445, 0.0248)
AIp1 (0.1649, 0.0549)
ORBvl6b (0.1743, 0.2800)
ORBvl6a (0.1542, 0.0060)
ORBvl5 (0.2362, 0.0109)
ORBvl2/3 (0.3735, 0.1228)
ORBvl1 (0.6395, 0.5835)
ORBm6a (0.5875, 0.1035)
ORBm5 (0.7017, 0.2567)
ORBm2/3 (0.9989, 0.5867)
ORBm1 (1.0000, 0.8552)
ORBl6a (0.3133, 0.0096)
ORBl5 (0.3252, 0.0109)
ORBl2/3 (0.4997, 0.1989)
ORBl1 (0.3684, 0.8950)
ILA6b (0.2148, 0.0210)
ILA6a (0.5364, 0.0857)
ILA5 (0.9002, 0.1497)
ILA2/3 (1.0000, 0.4651)
PL6b (0.1468, 0.0690)
PL6a (0.3220, 0.0885)
PL5 (0.5820, 0.1239)
PL2/3 (0.9895, 0.4651)
PL1 (0.9882, 0.8552)
ACAv6b (0.0586, 0.0156)
ACAv6a (0.1561, 0.0298)
ACAv5 (0.5267, 0.2215)
ACAv2/3 (0.4590, 0.4036)
ACAv1 (0.8726, 0.5513)
ACAd6b (0.0343, 0.0172)
ACAd6a (0.1122, 0.0172)
ACAd5 (0.4575, 0.0687)
ACAd2/3 (0.6636, 0.2255)
ACAd1 (0.7872, 0.6551)
VISpm6b (0.2520, 0.0703)
VISpm6a (0.1561, 0.0156)
VISpm5 (0.1561, 0.0247)
VISpm4 (0.1241, 0.0694)
VISpm2/3 (0.0607, 0.0461)
VISpm1 (0.0343, 0.0591)
VISpl6b (0.5519, 0.1600)
VISpl6a (0.3128, 0.0253)
VISpl5 (0.2181, 0.0232)
VISpl4 (0.1261, 0.0282)
VISpl2/3 (0.0343, 0.0092)
VISpl1 (0.0009, 0.0001)
VISp6b (0.1531, 0.0194)
VISp6a (0.1179, 0.0022)
VISp5 (0.0409, <0.0001)
VISp4 (0.0492, <0.0001)
VISp2/3 (0.0343, 0.0023)
VISp1 (0.0041, 0.0003)
VISl6b (0.2271, 0.0444)
VISl6a (0.2148, 0.0146)
VISl5 (0.1035, 0.0023)
VISl4 (0.1098, 0.0023)
VISl2/3 (0.0820, 0.0095)
VISl1 (0.0028, 0.0003)
VISam6b (0.1080, 0.0179)
VISam6a (0.0607, 0.0022)
VISam5 (0.0725, 0.0038)
VISam4 (0.0753, 0.0159)
VISam2/3 (0.0600, 0.0104)
VISal6b (0.2124, 0.0194)
VISal6a (0.3723, 0.0232)
VISal5 (0.1959, 0.0055)
VISal4 (0.3668, 0.0232)
VISal2/3 (0.0600, 0.0104)
VISal1 (0.0263, 0.0034)
AUDv6b (0.1056, 0.0050)
AUDv6a (0.1370, 0.0034)
AUDv5 (0.1447, 0.0070)
AUDv4 (0.3002, 0.0340)
AUDv2/3 (0.1179, 0.0130)
AUDv1 (0.0725, 0.1028)
AUDpo6b (0.3028, 0.0094)
AUDpo6a (0.0499, 0.0003)
AUDpo5 (0.1239, 0.0022)
AUDpo4 (0.1179, 0.0028)
AUDpo2/3 (0.1179, 0.0013)
AUDpo1 (0.0052, 0.0159)
AUDp6b (0.0343, <0.0001)
AUDp6a (0.0499, 0.0001)
AUDp5 (0.0725, 0.0009)
AUDp4 (0.2192, 0.0094)
AUDp2/3 (0.2147, 0.1483)
AUDp1 (0.0343, 0.0408)
AUDd6b (0.0409, 0.0001)
AUDd6a (0.0492, 0.0002)
AUDd5 (0.1179, 0.0028)
AUDd4 (0.2182, 0.0152)
AUDd2/3 (0.1730, 0.0945)
AUDd1 (0.0516, 0.0285)
VISC5 (0.1580, 0.0116)
VISC6b (0.1959, 0.1486)
VISC6a (0.1850, 0.0252)
VISC4 (0.2192, 0.0201)
VISC2/3 (0.3252, 0.0462)
VISC1 (0.1261, 0.0624)
GU6b (0.1292, 0.0282)
GU6a (0.1989, 0.0065)
GU5 (0.1959, 0.0044)
GU4 (0.1542, 0.0096)
GU2/3 (0.2149, 0.0221)
GU1 (0.1089, 0.0221)
0
10000
20000
30000
40000
c-Fos density

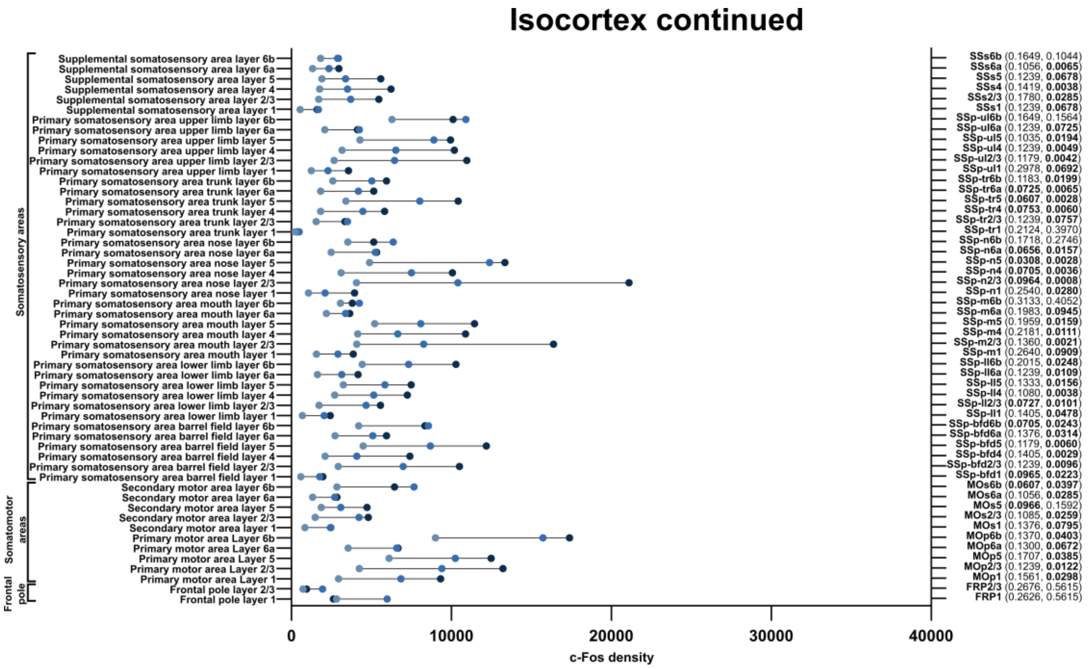
Isocortex continued
Somatosensory areas
Somatomotor areas
Frontal pole
Supplemental somatosensory area layer 6b
Supplemental somatosensory area layer 6a
Supplemental somatosensory area layer 5
Supplemental somatosensory area layer 4
Supplemental somatosensory area layer 2/3
Supplemental somatosensory area layer 1
Primary somatosensory area upper limb layer 6b
Primary somatosensory area upper limb layer 6a
Primary somatosensory area upper limb layer 5
Primary somatosensory area upper limb layer 4
Primary somatosensory area upper limb layer 2/3
Primary somatosensory area upper limb layer 1
Primary somatosensory area trunk layer 6b
Primary somatosensory area trunk layer 6a
Primary somatosensory area trunk layer 5
Primary somatosensory area trunk layer 4
Primary somatosensory area trunk layer 2/3
Primary somatosensory area trunk layer 1
Primary somatosensory area nose layer 6b
Primary somatosensory area nose layer 6a
Primary somatosensory area nose layer 5
Primary somatosensory area nose layer 4
Primary somatosensory area nose layer 2/3
Primary somatosensory area nose layer 1
Primary somatosensory area mouth layer 6b
Primary somatosensory area mouth layer 6a
Primary somatosensory area mouth layer 5
Primary somatosensory area mouth layer 4
Primary somatosensory area mouth layer 2/3
Primary somatosensory area mouth layer 1
Primary somatosensory area lower limb layer 6b
Primary somatosensory area lower limb layer 6a
Primary somatosensory area lower limb layer 5
Primary somatosensory area lower limb layer 4
Primary somatosensory area lower limb layer 2/3
Primary somatosensory area lower limb layer 1
Primary somatosensory area barrel field layer 6b
Primary somatosensory area barrel field layer 6a
Primary somatosensory area barrel field layer 5
Primary somatosensory area barrel field layer 4
Primary somatosensory area barrel field layer 2/3
Primary somatosensory area barrel field layer 1
Secondary motor area layer 6b
Secondary motor area layer 6a
Secondary motor area layer 5
Secondary motor area layer 2/3
Secondary motor area layer 1
Primary motor area Layer 6b
Primary motor area Layer 6a
Primary motor area Layer 5
Primary motor area Layer 2/3
Primary motor area Layer 1
Frontal pole layer 2/3
Frontal pole layer 1
SSs6b (0.1649, 0.1044)
SSs6a (0.1056, **0.0065**)
SSs5 (0.1239, **0.0678**)
SSs4 (0.1419, **0.0038**)
SSs2/3 (0.1780, **0.0285**)
SSs1 (0.1239, **0.0678**)
SSp-ul6b (0.1649, 0.1564)
SSp-ul6a (0.1239, **0.0725**)
SSp-ul5 (0.1035, **0.0194**)
SSp-ul4 (0.1239, **0.0049**)
SSp-ul2/3 (0.1179, **0.0042**)
SSp-ul1 (0.2978, **0.0692**)
SSp-tr6b (0.1183, **0.0199**)
SSp-tr6a (**0.0725**, **0.0065**)
SSp-tr5 (**0.0607**, **0.0028**)
SSp-tr4 (**0.0753**, **0.0060**)
SSp-tr2/3 (0.1239, **0.0757**)
SSp-tr1 (0.2124, 0.3970)
SSp-n6b (0.1718, 0.2746)
SSp-n6a (**0.0656**, **0.0157**)
SSp-n5 (**0.0308**, **0.0028**)
SSp-n4 (**0.0705**, **0.0036**)
SSp-n2/3 (**0.0964**, **0.0008**)
SSp-n1 (0.2540, **0.0280**)
SSp-m6b (0.3133, 0.4052)
SSp-m6a (0.1983, **0.0945**)
SSp-m5 (0.1959, **0.0159**)
SSp-m4 (0.2181, **0.0111**)
SSp-m2/3 (0.1360, **0.0021**)
SSp-m1 (0.2640, **0.0909**)
SSp-ll6b (0.2015, **0.0248**)
SSp-ll6a (0.1239, **0.0109**)
SSp-ll5 (0.1333, **0.0156**)
SSp-ll4 (0.1080, **0.0038**)
SSp-ll2/3 (**0.0727**, **0.0101**)
SSp-ll1 (0.1405, **0.0478**)
SSp-bfd6b (**0.0705**, **0.0243**)
SSp-bfd6a (0.1376, **0.0314**)
SSp-bfd5 (0.1179, **0.0060**)
SSp-bfd4 (0.1405, **0.0029**)
SSp-bfd2/3 (0.1239, **0.0096**)
SSp-bfd1 (**0.0965**, **0.0223**)
MOs6b (**0.0607**, **0.0397**)
MOs6a (0.1056, **0.0285**)
MOs5 (**0.0966**, 0.1592)
MOs2/3 (0.1085, **0.0259**)
MOs1 (0.1376, **0.0795**)
MOp6b (0.1370, **0.0403**)
MOp6a (0.1300, **0.0672**)
MOp5 (0.1707, **0.0385**)
MOp2/3 (0.1239, **0.0122**)
MOp1 (0.1561, **0.0298**)
FRP2/3 (0.2676, 0.5615)
FRP1 (0.2626, 0.5615)
0
10000
20000
30000
40000
c-Fos density

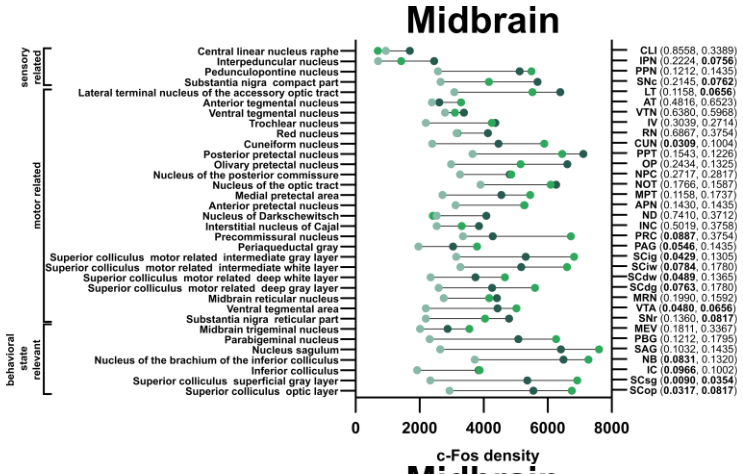
Midbrain
sensory related
motor related
behavioral state relevant
Central linear nucleus raphe
Interpeduncular nucleus
Pedunculopontine nucleus
Substantia nigra compact part
Lateral terminal nucleus of the accessory optic tract
Anterior tegmental nucleus
Ventral tegmental nucleus
Trochlear nucleus
Red nucleus
Cuneiform nucleus
Posterior pretectal nucleus
Olivary pretectal nucleus
Nucleus of the posterior commissure
Nucleus of the optic tract
Medial pretectal area
Anterior pretectal nucleus
Nucleus of Darkschewitsch
Interstitial nucleus of Cajal
Precommissural nucleus
Periaqueductal gray
Superior colliculus motor related intermediate gray layer
Superior colliculus motor related intermediate white layer
Superior colliculus motor related deep white layer
Superior colliculus motor related deep gray layer
Midbrain reticular nucleus
Ventral tegmental area
Substantia nigra reticular part
Midbrain trigeminal nucleus
Parabigeminal nucleus
Nucleus sagulum
Nucleus of the brachium of the inferior colliculus
Inferior colliculus
Superior colliculus superficial gray layer
Superior colliculus optic layer
CLI (0.8558, 0.3389)
IPN (0.2224, **0.0756**)
PPN (0.1212, 0.1435)
SNc (0.2145, **0.0762**)
LT (0.1158, **0.0656**)
AT (0.4816, 0.6523)
VTN (0.6380, 0.5968)
IV (0.3039, 0.2714)
RN (0.6867, 0.3754)
CUN (**0.0309**, 0.1004)
PPT (0.1543, 0.1226)
OP (0.2434, 0.1325)
NPC (0.2717, 0.2817)
NOT (0.1766, 0.1587)
MPT (0.1158, 0.1737)
APN (0.1430, 0.1435)
ND (0.7410, 0.3712)
INC (0.5019, 0.3758)
PRC (**0.0887**, 0.3754)
PAG (**0.0546**, 0.1435)
SCig (**0.0429**, 0.1305)
SCiw (**0.0784**, 0.1780)
SCdw (**0.0489**, 0.1365)
SCdg (**0.0763**, 0.1780)
MRN (0.1990, 0.1592)
VTA (**0.0480**, **0.0656**)
SNr (0.1360, **0.0817**)
MEV (0.1811, 0.3367)
PBG (0.1212, 0.1795)
SAG (0.1032, 0.1435)
NB (**0.0831**, 0.1320)
IC (**0.0966**, 0.1002)
SCsg (**0.0090**, **0.0354**)
SCop (**0.0317**, **0.0817**)
0
2000
4000
6000
8000
c-Fos density

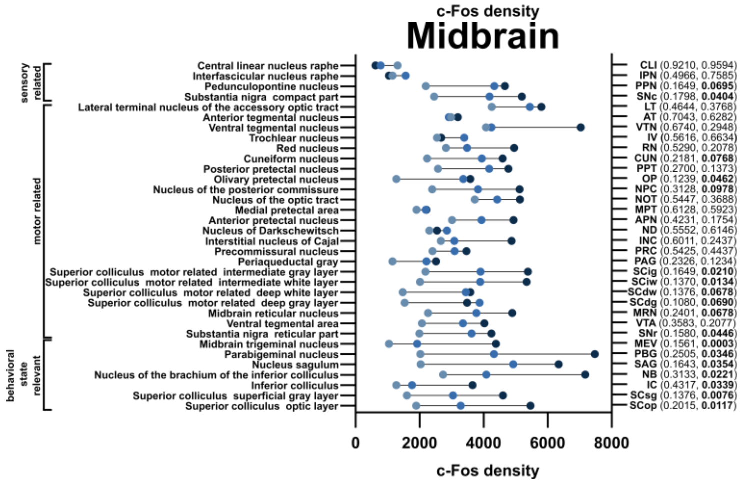
Midbrain
sensory related
motor related
behavioral state relevant
Central linear nucleus raphe
Interfascicular nucleus raphe
Pedunculopontine nucleus
Substantia nigra compact part
Lateral terminal nucleus of the accessory optic tract
Anterior tegmental nucleus
Ventral tegmental nucleus
Trochlear nucleus
Red nucleus
Cuneiform nucleus
Posterior pretectal nucleus
Olivary pretectal nucleus
Nucleus of the posterior commissure
Nucleus of the optic tract
Medial pretectal area
Anterior pretectal nucleus
Nucleus of Darkschewitsch
Interstitial nucleus of Cajal
Precommissural nucleus
Periaqueductal gray
Superior colliculus motor related intermediate gray layer
Superior colliculus motor related intermediate white layer
Superior colliculus motor related deep white layer
Superior colliculus motor related deep gray layer
Midbrain reticular nucleus
Ventral tegmental area
Substantia nigra reticular part
Midbrain trigeminal nucleus
Parabigeminal nucleus
Nucleus sagulum
Nucleus of the brachium of the inferior colliculus
Inferior colliculus
Superior colliculus superficial gray layer
Superior colliculus optic layer
CLI (0.9210, 0.9594)
IPN (0.4966, 0.7585)
PPN (0.1649, **0.0695**)
SNc (0.1798, **0.0404**)
LT (0.4644, 0.3768)
AT (0.7043, 0.6282)
VTN (0.6740, 0.2948)
IV (0.5616, 0.6634)
RN (0.5290, 0.2078)
CUN (0.2181, **0.0768**)
PPT (0.2700, 0.1373)
OP (0.1239, **0.0462**)
NPC (0.3128, **0.0978**)
NOT (0.5447, 0.3688)
MPT (0.6128, 0.5923)
APN (0.4231, 0.1754)
ND (0.5552, 0.6146)
INC (0.6011, 0.2437)
PRC (0.5425, 0.4437)
PAG (0.2326, 0.1234)
SCig (0.1649, **0.0210**)
SCiw (0.1370, **0.0134**)
SCdw (0.1376, **0.0678**)
SCdg (0.1080, **0.0690**)
MRN (0.2401, **0.0678**)
VTA (0.3583, 0.2077)
SNr (0.1580, **0.0446**)
MEV (0.1561, **0.0003**)
PBG (0.2505, **0.0346**)
SAG (0.1643, **0.0354**)
NB (0.3133, **0.0221**)
IC (0.4317, **0.0339**)
SCsg (0.1376, **0.0076**)
SCop (0.2015, **0.0117**)
0
2000
4000
6000
8000
c-Fos density

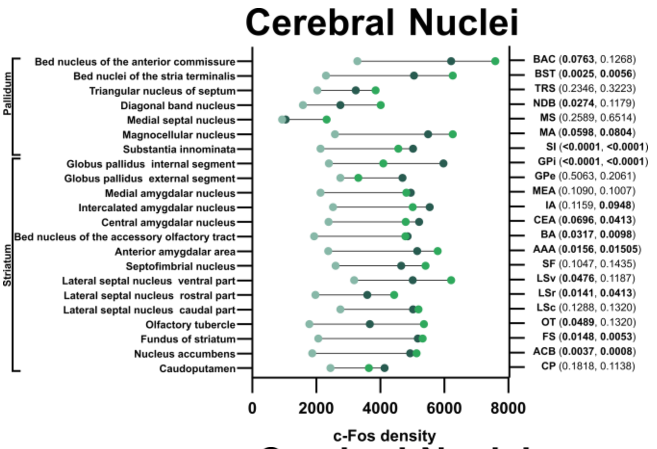
Cerebral Nuclei
Pallidum
Striatum
Bed nucleus of the anterior commissure
Bed nuclei of the stria terminalis
Triangular nucleus of septum
Diagonal band nucleus
Medial septal nucleus
Magnocellular nucleus
Substantia innominata
Globus pallidus internal segment
Globus pallidus external segment
Medial amygdalar nucleus
Intercalated amygdalar nucleus
Central amygdalar nucleus
Bed nucleus of the accessory olfactory tract
Anterior amygdalar area
Septofimbrial nucleus
Lateral septal nucleus ventral part
Lateral septal nucleus rostral part
Lateral septal nucleus caudal part
Olfactory tubercle
Fundus of striatum
Nucleus accumbens
Caudoputamen
BAC (0.0763, 0.1268)
BST (0.0025, 0.0056)
TRS (0.2346, 0.3223)
NDB (0.0274, 0.1179)
MS (0.2589, 0.6514)
MA (0.0598, 0.0804)
SI (<0.0001, <0.0001)
GPi (<0.0001, <0.0001)
GPe (0.5063, 0.2061)
MEA (0.1090, 0.1007)
IA (0.1159, 0.0948)
CEA (0.0696, 0.0413)
BA (0.0317, 0.0098)
AAA (0.0156, 0.01505)
SF (0.1047, 0.1435)
LSv (0.0476, 0.1187)
LSr (0.0141, 0.0413)
LSc (0.1288, 0.1320)
OT (0.0489, 0.1320)
FS (0.0148, 0.0053)
ACB (0.0037, 0.0008)
CP (0.1818, 0.1138)
0
2000
4000
6000
8000
c-Fos density

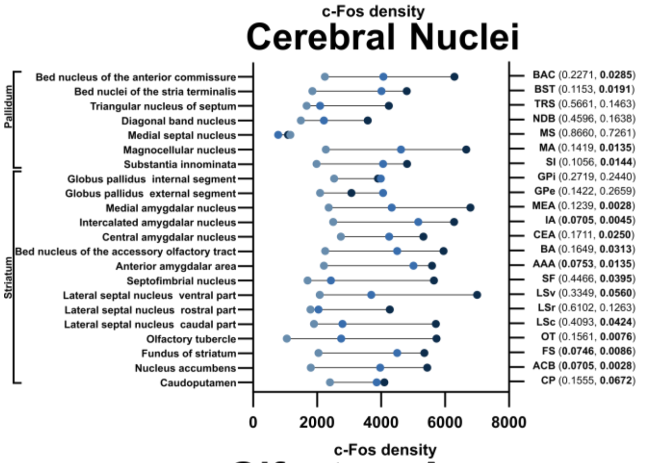
Cerebral Nuclei
Pallidum
Striatum
Bed nucleus of the anterior commissure
Bed nuclei of the stria terminalis
Triangular nucleus of septum
Diagonal band nucleus
Medial septal nucleus
Magnocellular nucleus
Substantia innominata
Globus pallidus internal segment
Globus pallidus external segment
Medial amygdalar nucleus
Intercalated amygdalar nucleus
Central amygdalar nucleus
Bed nucleus of the accessory olfactory tract
Anterior amygdalar area
Septofimbrial nucleus
Lateral septal nucleus ventral part
Lateral septal nucleus rostral part
Lateral septal nucleus caudal part
Olfactory tubercle
Fundus of striatum
Nucleus accumbens
Caudoputamen
BAC (0.2271, 0.0285)
BST (0.1153, 0.0191)
TRS (0.5661, 0.1463)
NDB (0.4596, 0.1638)
MS (0.8660, 0.7261)
MA (0.1419, 0.0135)
SI (0.1056, 0.0144)
GPi (0.2719, 0.2440)
GPe (0.1422, 0.2659)
MEA (0.1239, 0.0028)
IA (0.0705, 0.0045)
CEA (0.1711, 0.0250)
BA (0.1649, 0.0313)
AAA (0.0753, 0.0135)
SF (0.4466, 0.0395)
LSv (0.3349, 0.0560)
LSr (0.6102, 0.1263)
LSc (0.4093, 0.0424)
OT (0.1561, 0.0076)
FS (0.0746, 0.0086)
ACB (0.0705, 0.0028)
CP (0.1555, 0.0672)
0
2000
4000
6000
8000
c-Fos density

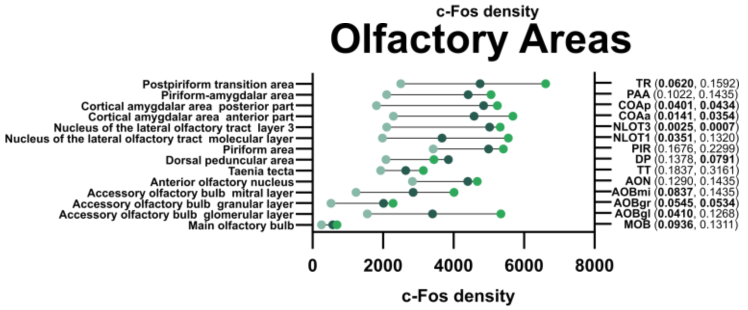
Olfactory Areas
Postpiriform transition area
Piriform-amygdalar area
Cortical amygdalar area posterior part
Cortical amygdalar area anterior part
Nucleus of the lateral olfactory tract layer 3
Nucleus of the lateral olfactory tract molecular layer
Piriform area
Dorsal peduncular area
Taenia tecta
Anterior olfactory nucleus
Accessory olfactory bulb mitral layer
Accessory olfactory bulb granular layer
Accessory olfactory bulb glomerular layer
Main olfactory bulb
TR (0.0620, 0.1592)
PAA (0.1022, 0.1435)
COAp (0.0401, 0.0434)
COAa (0.0141, 0.0354)
NLOT3 (0.0025, 0.0007)
NLOT1 (0.0351, 0.1320)
PIR (0.1676, 0.2299)
DP (0.1378, 0.0791)
TT (0.1837, 0.3161)
AON (0.1290, 0.1435)
AOBmi (0.0837, 0.1435)
AOBgr (0.0545, 0.0534)
AOBgl (0.0410, 0.1268)
MOB (0.0936, 0.1311)
0
2000
4000
6000
8000
c-Fos density

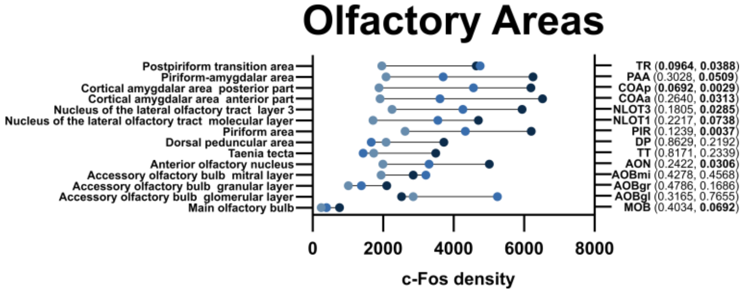
Olfactory Areas
Postpiriform transition area
Piriform-amygdalar area
Cortical amygdalar area posterior part
Cortical amygdalar area anterior part
Nucleus of the lateral olfactory tract layer 3
Nucleus of the lateral olfactory tract molecular layer
Piriform area
Dorsal peduncular area
Taenia tecta
Anterior olfactory nucleus
Accessory olfactory bulb mitral layer
Accessory olfactory bulb granular layer
Accessory olfactory bulb glomerular layer
Main olfactory bulb
TR (0.0964, 0.0388)
PAA (0.3028, 0.0509)
COAp (0.0692, 0.0029)
COAa (0.2640, 0.0313)
NLOT3 (0.1805, 0.0285)
NLOT1 (0.2217, 0.0738)
PIR (0.1239, 0.0037)
DP (0.8629, 0.2192)
TT (0.8171, 0.2339)
AON (0.2422, 0.0306)
AOBmi (0.4278, 0.4568)
AOBgr (0.4786, 0.1686)
AOBgl (0.3165, 0.7655)
MOB (0.4034, 0.0692)
0
2000
4000
6000
8000
c-Fos density

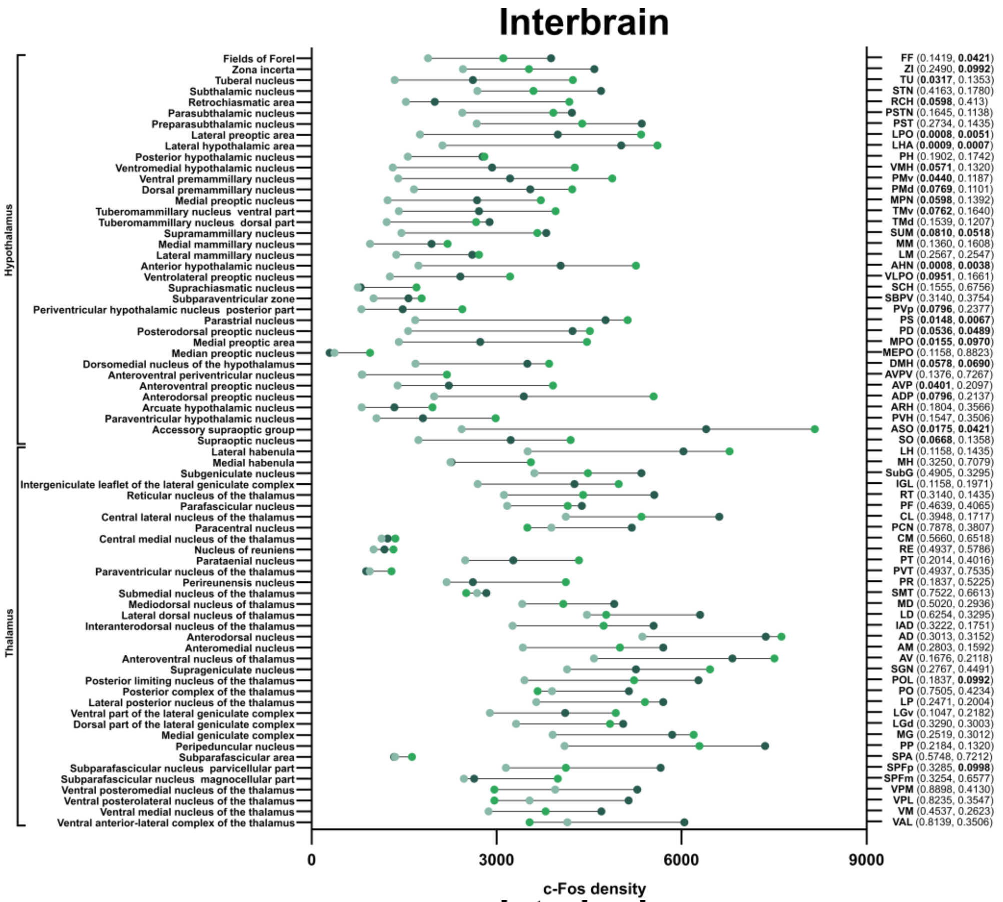
Interbrain
Hypothalamus
Thalamus
Fields of Forel
Zona incerta
Tuberal nucleus
Subthalamic nucleus
Retrochiasmatic area
Parasubthalamic nucleus
Preparasubthalamic nucleus
Lateral preoptic area
Lateral hypothalamic area
Posterior hypothalamic nucleus
Ventromedial hypothalamic nucleus
Ventral premammillary nucleus
Dorsal premammillary nucleus
Medial preoptic nucleus
Tuberomammillary nucleus ventral part
Tuberomammillary nucleus dorsal part
Supramammillary nucleus
Medial mammillary nucleus
Lateral mammillary nucleus
Anterior hypothalamic nucleus
Ventrolateral preoptic nucleus
Suprachiasmatic nucleus
Subparaventricular zone
Periventricular hypothalamic nucleus posterior part
Parastrial nucleus
Posterodorsal preoptic nucleus
Medial preoptic area
Median preoptic nucleus
Dorsomedial nucleus of the hypothalamus
Anteroventral periventricular nucleus
Anteroventral preoptic nucleus
Anterodorsal preoptic nucleus
Arcuate hypothalamic nucleus
Paraventricular hypothalamic nucleus
Accessory supraoptic group
Supraoptic nucleus
Lateral habenula
Medial habenula
Subgeniculate nucleus
Intergeniculate leaflet of the lateral geniculate complex
Reticular nucleus of the thalamus
Parafascicular nucleus
Central lateral nucleus of the thalamus
Paracentral nucleus
Central medial nucleus of the thalamus
Nucleus of reuniens
Parataenial nucleus
Paraventricular nucleus of the thalamus
Perireunensis nucleus
Submedial nucleus of the thalamus
Mediodorsal nucleus of thalamus
Lateral dorsal nucleus of thalamus
Interanterodorsal nucleus of the thalamus
Anterodorsal nucleus
Anteromedial nucleus
Anteroventral nucleus of thalamus
Suprageniculate nucleus
Posterior limiting nucleus of the thalamus
Posterior complex of the thalamus
Lateral posterior nucleus of the thalamus
Ventral part of the lateral geniculate complex
Dorsal part of the lateral geniculate complex
Medial geniculate complex
Peripeduncular nucleus
Subparafascicular area
Subparafascicular nucleus parvicellular part
Subparafascicular nucleus magnocellular part
Ventral posteromedial nucleus of the thalamus
Ventral posterolateral nucleus of the thalamus
Ventral medial nucleus of the thalamus
Ventral anterior-lateral complex of the thalamus
FF (0.1419, **0.0421**)
ZI (0.2490, **0.0992**)
TU (**0.0317**, 0.1353)
STN (0.4163, 0.1780)
RCH (**0.0598**, 0.413)
PSTN (0.1645, 0.1138)
PST (0.2734, 0.1435)
LPO (**0.0008**, **0.0051**)
LHA (**0.0009**, **0.0007**)
PH (0.1902, 0.1742)
VMH (**0.0571**, 0.1320)
PMv (**0.0440**, 0.1187)
PMd (**0.0769**, 0.1101)
MPN (**0.0598**, 0.1392)
TMv (**0.0762**, 0.1640)
TMd (0.1539, 0.1207)
SUM (**0.0810**, **0.0518**)
MM (0.1360, 0.1608)
LM (0.2567, 0.2547)
AHN (**0.0008**, **0.0038**)
VLPO (**0.0951**, 0.1661)
SCH (0.1555, 0.6756)
SBPV (0.3140, 0.3754)
PVp (**0.0796**, 0.2377)
PS (**0.0148**, **0.0067**)
PD (**0.0536**, **0.0489**)
MPO (**0.0155**, **0.0970**)
MEPO (0.1158, 0.8823)
DMH (**0.0578**, **0.0690**)
AVPV (0.1376, 0.7267)
AVP (**0.0401**, 0.2097)
ADP (**0.0796**, 0.2137)
ARH (0.1804, 0.3566)
PVH (0.1547, 0.3506)
ASO (**0.0175**, **0.0421**)
SO (**0.0668**, 0.1358)
LH (0.1158, 0.1435)
MH (0.3250, 0.7079)
SubG (0.4905, 0.3295)
IGL (0.1158, 0.1971)
RT (0.3140, 0.1435)
PF (0.4639, 0.4065)
CL (0.3948, 0.1717)
PCN (0.7878, 0.3807)
CM (0.5660, 0.6518)
RE (0.4937, 0.5786)
PT (0.2014, 0.4016)
PVT (0.4937, 0.7535)
PR (0.1837, 0.5225)
SMT (0.7522, 0.6613)
MD (0.5020, 0.2936)
LD (0.6254, 0.3295)
IAD (0.3222, 0.1751)
AD (0.3013, 0.3152)
AM (0.2803, 0.1592)
AV (0.1676, 0.2118)
SGN (0.2767, 0.4491)
POL (0.1837, **0.0992**)
PO (0.7505, 0.4234)
LP (0.2471, 0.2004)
LGv (0.1047, 0.2182)
LGd (0.3290, 0.3003)
MG (0.2519, 0.3012)
PP (0.2184, 0.1320)
SPA (0.5748, 0.7212)
SPFp (0.3285, **0.0998**)
SPFm (0.3254, 0.6577)
VPM (0.8898, 0.4130)
VPL (0.8235, 0.3547)
VM (0.4537, 0.2623)
VAL (0.8139, 0.3506)
0
3000
6000
9000
c-Fos density

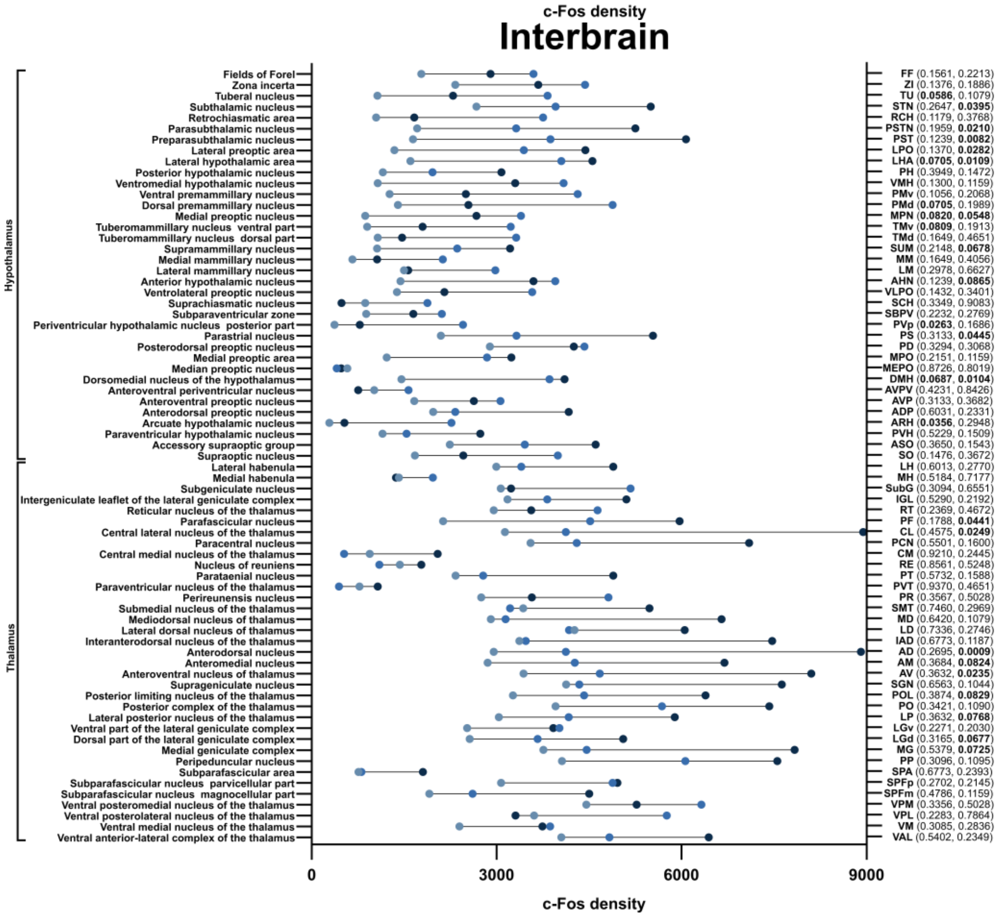
Interbrain
Hypothalamus
Thalamus
Fields of Forel
Zona incerta
Tuberal nucleus
Subthalamic nucleus
Retrochiasmatic area
Parasubthalamic nucleus
Preparasubthalamic nucleus
Lateral preoptic area
Lateral hypothalamic area
Posterior hypothalamic nucleus
Ventromedial hypothalamic nucleus
Ventral premammillary nucleus
Dorsal premammillary nucleus
Medial preoptic nucleus
Tuberomammillary nucleus ventral part
Tuberomammillary nucleus dorsal part
Supramammillary nucleus
Medial mammillary nucleus
Lateral mammillary nucleus
Anterior hypothalamic nucleus
Ventrolateral preoptic nucleus
Suprachiasmatic nucleus
Subparaventricular zone
Periventricular hypothalamic nucleus posterior part
Parastrial nucleus
Posterodorsal preoptic nucleus
Medial preoptic area
Median preoptic nucleus
Dorsomedial nucleus of the hypothalamus
Anteroventral periventricular nucleus
Anteroventral preoptic nucleus
Anterodorsal preoptic nucleus
Arcuate hypothalamic nucleus
Paraventricular hypothalamic nucleus
Accessory supraoptic group
Supraoptic nucleus
Lateral habenula
Medial habenula
Subgeniculate nucleus
Intergeniculate leaflet of the lateral geniculate complex
Reticular nucleus of the thalamus
Parafascicular nucleus
Central lateral nucleus of the thalamus
Paracentral nucleus
Central medial nucleus of the thalamus
Nucleus of reuniens
Parataenial nucleus
Paraventricular nucleus of the thalamus
Perireunensis nucleus
Submedial nucleus of the thalamus
Mediodorsal nucleus of thalamus
Lateral dorsal nucleus of thalamus
Interanterodorsal nucleus of the thalamus
Anterodorsal nucleus
Anteromedial nucleus
Anteroventral nucleus of thalamus
Suprageniculate nucleus
Posterior limiting nucleus of the thalamus
Posterior complex of the thalamus
Lateral posterior nucleus of the thalamus
Ventral part of the lateral geniculate complex
Dorsal part of the lateral geniculate complex
Medial geniculate complex
Peripeduncular nucleus
Subparafascicular area
Subparafascicular nucleus parvicellular part
Subparafascicular nucleus magnocellular part
Ventral posteromedial nucleus of the thalamus
Ventral posterolateral nucleus of the thalamus
Ventral medial nucleus of the thalamus
Ventral anterior-lateral complex of the thalamus
FF (0.1561, 0.2213)
ZI (0.1376, 0.1886)
TU (**0.0586**, 0.1079)
STN (0.2647, **0.0395**)
RCH (0.1179, 0.3768)
PSTN (0.1959, **0.0210**)
PST (0.1239, **0.0082**)
LPO (0.1370, **0.0282**)
LHA (**0.0705**, **0.0109**)
PH (0.3949, 0.1472)
VMH (0.1300, 0.1159)
PMv (0.1056, 0.2068)
PMd (**0.0705**, 0.1989)
MPN (**0.0820**, **0.0548**)
TMv (**0.0809**, 0.1913)
TMd (0.1649, 0.4651)
SUM (0.2148, **0.0678**)
MM (0.1649, 0.4056)
LM (0.2978, 0.6627)
AHN (0.1239, **0.0865**)
VLPO (0.1432, 0.3401)
SCH (0.3349, 0.9083)
SBPV (0.2232, 0.2769)
PVp (**0.0263**, 0.1686)
PS (0.3133, **0.0445**)
PD (0.3294, 0.3068)
MPO (0.2151, 0.1159)
MEPO (0.8726, 0.8019)
DMH (**0.0687**, **0.0104**)
AVPV (0.4231, 0.8426)
AVP (0.3133, 0.3682)
ADP (0.6031, 0.2331)
ARH (**0.0356**, 0.2948)
PVH (0.5229, 0.1509)
ASO (0.3650, 0.1543)
SO (0.1476, 0.3672)
LH (0.6013, 0.2770)
MH (0.5184, 0.7177)
SubG (0.3094, 0.6551)
IGL (0.5290, 0.2192)
RT (0.2369, 0.4672)
PF (0.1788, **0.0441**)
CL (0.4575, **0.0249**)
PCN (0.5501, 0.1600)
CM (0.9210, 0.2445)
RE (0.8561, 0.5248)
PT (0.5732, 0.1588)
PVT (0.9370, 0.4651)
PR (0.3567, 0.5028)
SMT (0.7460, 0.2969)
MD (0.6420, 0.1079)
LD (0.7336, 0.2746)
IAD (0.6773, 0.1187)
AD (0.2695, **0.0009**)
AM (0.3684, **0.0824**)
AV (0.3632, **0.0235**)
SGN (0.6563, 0.1044)
POL (0.3874, **0.0829**)
PO (0.3421, 0.1090)
LP (0.3632, **0.0768**)
LGv (0.2271, 0.2030)
LGd (0.3165, **0.0677**)
MG (0.5379, **0.0725**)
PP (0.3096, 0.1095)
SPA (0.6773, 0.2393)
SPFp (0.2702, 0.2145)
SPFm (0.4786, 0.1159)
VPM (0.3356, 0.5028)
VPL (0.2283, 0.7864)
VM (0.3085, 0.2836)
VAL (0.5402, 0.2349)
0
3000
6000
9000
c-Fos density

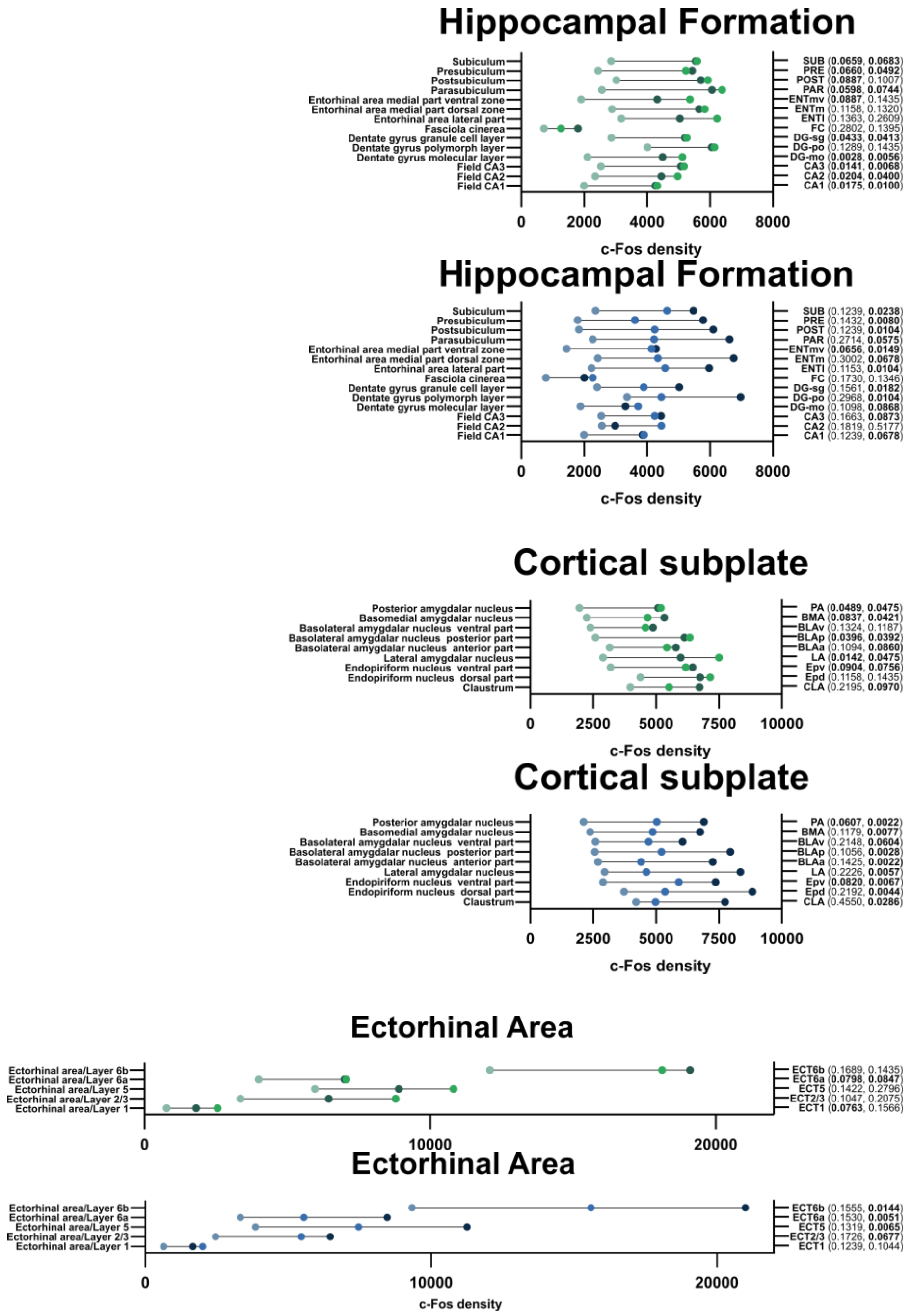
Hippocampal Formation
Subiculum
Presubiculum
Postsubiculum
Parasubiculum
Entorhinal area medial part ventral zone
Entorhinal area medial part dorsal zone
Entorhinal area lateral part
Fasciola cinerea
Dentate gyrus granule cell layer
Dentate gyrus polymorph layer
Dentate gyrus molecular layer
Field CA3
Field CA2
Field CA1
SUB (0.0659, 0.0683)
PRE (0.0660, 0.0492)
POST (0.0887, 0.1007)
PAR (0.0598, 0.0744)
ENTmv (0.0887, 0.1435)
ENTm (0.1158, 0.1320)
ENTl (0.1363, 0.2609)
FC (0.2802, 0.1395)
DG-sg (0.0433, 0.0413)
DG-po (0.1289, 0.1435)
DG-mo (0.0028, 0.0056)
CA3 (0.0141, 0.0068)
CA2 (0.0204, 0.0400)
CA1 (0.0175, 0.0100)
0 2000 4000 6000 8000
c-Fos density
Hippocampal Formation
Subiculum
Presubiculum
Postsubiculum
Parasubiculum
Entorhinal area medial part ventral zone
Entorhinal area medial part dorsal zone
Entorhinal area lateral part
Fasciola cinerea
Dentate gyrus granule cell layer
Dentate gyrus polymorph layer
Dentate gyrus molecular layer
Field CA3
Field CA2
Field CA1
SUB (0.1239, 0.0238)
PRE (0.1432, 0.0080)
POST (0.1239, 0.0104)
PAR (0.2714, 0.0575)
ENTmv (0.0656, 0.0149)
ENTm (0.3002, 0.0678)
ENTl (0.1153, 0.0104)
FC (0.1730, 0.1346)
DG-sg (0.1561, 0.0182)
DG-po (0.2968, 0.0104)
DG-mo (0.1098, 0.0868)
CA3 (0.1663, 0.0873)
CA2 (0.1819, 0.5177)
CA1 (0.1239, 0.0678)
0 2000 4000 6000 8000
c-Fos density
Cortical subplate
Posterior amygdalar nucleus
Basomedial amygdalar nucleus
Basolateral amygdalar nucleus ventral part
Basolateral amygdalar nucleus posterior part
Basolateral amygdalar nucleus anterior part
Lateral amygdalar nucleus
Endopiriform nucleus ventral part
Endopiriform nucleus dorsal part
Claustrum
PA (0.0489, 0.0475)
BMA (0.0837, 0.0421)
BLAv (0.1324, 0.1187)
BLAp (0.0396, 0.0392)
BLAa (0.1094, 0.0860)
LA (0.0142, 0.0475)
Epv (0.0904, 0.0756)
Epd (0.1158, 0.1435)
CLA (0.2195, 0.0970)
0 2500 5000 7500 10000
c-Fos density
Cortical subplate
Posterior amygdalar nucleus
Basomedial amygdalar nucleus
Basolateral amygdalar nucleus ventral part
Basolateral amygdalar nucleus posterior part
Basolateral amygdalar nucleus anterior part
Lateral amygdalar nucleus
Endopiriform nucleus ventral part
Endopiriform nucleus dorsal part
Claustrum
PA (0.0607, 0.0022)
BMA (0.1179, 0.0077)
BLAv (0.2148, 0.0604)
BLAp (0.1056, 0.0028)
BLAa (0.1425, 0.0022)
LA (0.2226, 0.0057)
Epv (0.0820, 0.0067)
Epd (0.2192, 0.0044)
CLA (0.4550, 0.0286)
0 2500 5000 7500 10000
c-Fos density
Ectorhinal Area
Ectorhinal area/Layer 6b
Ectorhinal area/Layer 6a
Ectorhinal area/Layer 5
Ectorhinal area/Layer 2/3
Ectorhinal area/Layer 1
ECT6b (0.1689, 0.1435)
ECT6a (0.0798, 0.0847)
ECT5 (0.1422, 0.2796)
ECT2/3 (0.1047, 0.2075)
ECT1 (0.0763, 0.1566)
0 10000 20000
Ectorhinal Area
Ectorhinal area/Layer 6b
Ectorhinal area/Layer 6a
Ectorhinal area/Layer 5
Ectorhinal area/Layer 2/3
Ectorhinal area/Layer 1
ECT6b (0.1555, 0.0144)
ECT6a (0.1530, 0.0051)
ECT5 (0.1319, 0.0065)
ECT2/3 (0.1726, 0.0677)
ECT1 (0.1239, 0.1044)
0 10000 20000
c-Fos density

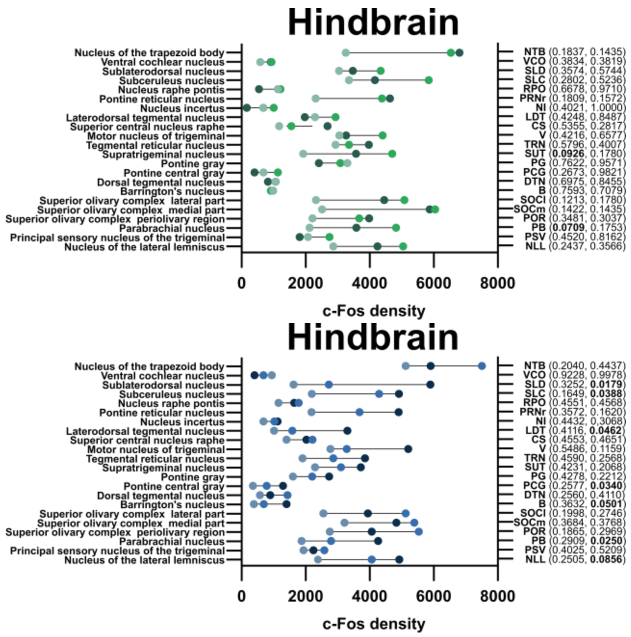

Fig. Supp. 3: **c-Fos signal across the brain after sugar and cocaine exposures.**
Green colors correspond to the mean signal from mice after sucrose treatment (light - Water, medium - Sugar 1 d, dark - Sugar 7 d) and blue colors correspond to cocaine treatment (light - Saline, medium - Cocaine 1 d, dark - Cocaine 7 d). On the left axis are the names of the structures and on the right are corresponding abbreviations and statistical differences among groups in brackets. In brackets for natural (Water vs. Sugar 1 d, Water vs. Sugar 7 d) and for addictive (Saline vs. Cocaine 1 d, Saline vs. Cocaine 7 d). Statistical differences of $p<0.1$ were labeled with a bold font.
P-value was calculated for each structure in the following steps: first, a generalized linear model (GLM) was calculated. Then, for each GLM a Dunnett's test was performed. Finally, due to a large number of structures, a Benjamin-Hochberg false discovery rate correction was performed on p-values with a 0.1 cut-off. N Water=7, N Sugar 1 d=5, N Sugar 7 d=7, N Saline=7, N Cocaine 1 d=6, N Cocaine 7 d=6.

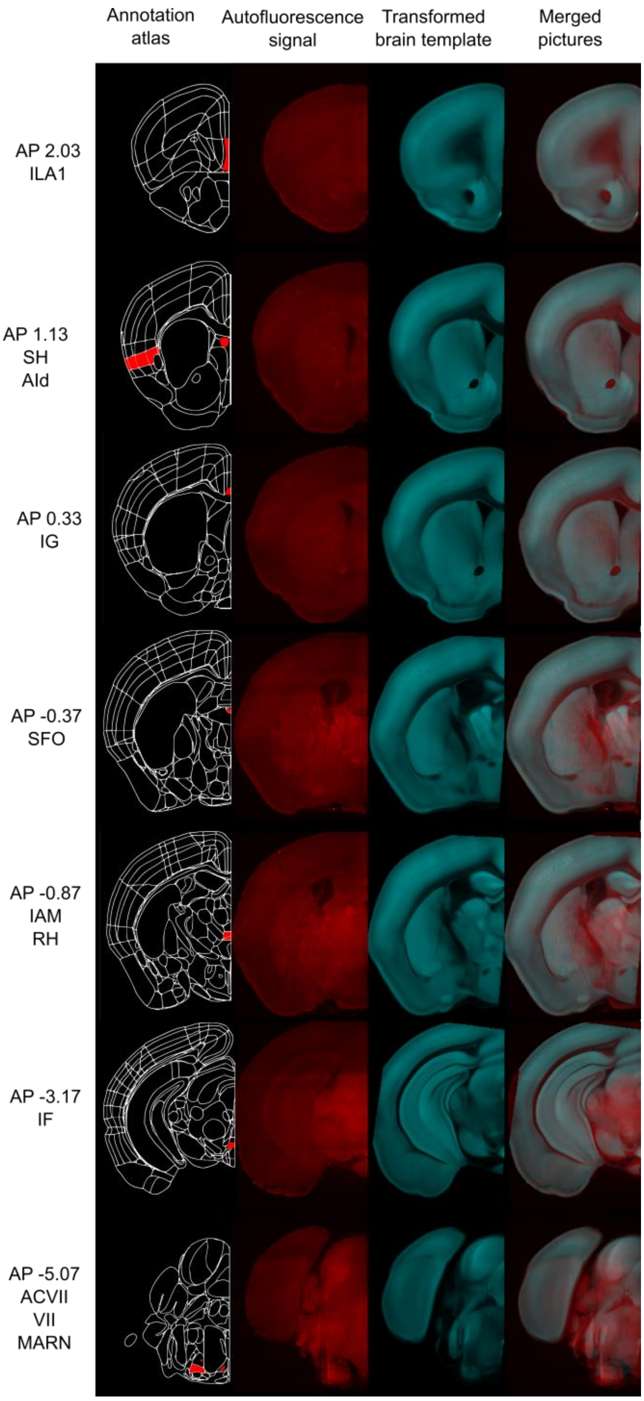

Fig. Supp. 4: Alignment of the brain template to the autofluorescence image. During the alignment process, the brain template from the Allen Brain Institute was transformed to fit the autofluorescence images of collected brains. Several brain regions (marked with red on the Annotation atlas) were not determined by the ClearMap software. Thus, these structures were excluded from further analysis. Interfascicular nucleus raphe (IF), oculomotor nucleus (III), subfornical organ (SFO), rhomboid nucleus (RH), interanteromedial nucleus of the thalamus (IAM), induseum griseum (IG), magnocellular reticular nucleus (MARN), accessory facial motor nucleus (ACVII), facial motor nucleus (VII), septohippocampal nucleus (SH), infralimbic area layer 1 (ILA1), agranular insular area, dorsal part (AId).

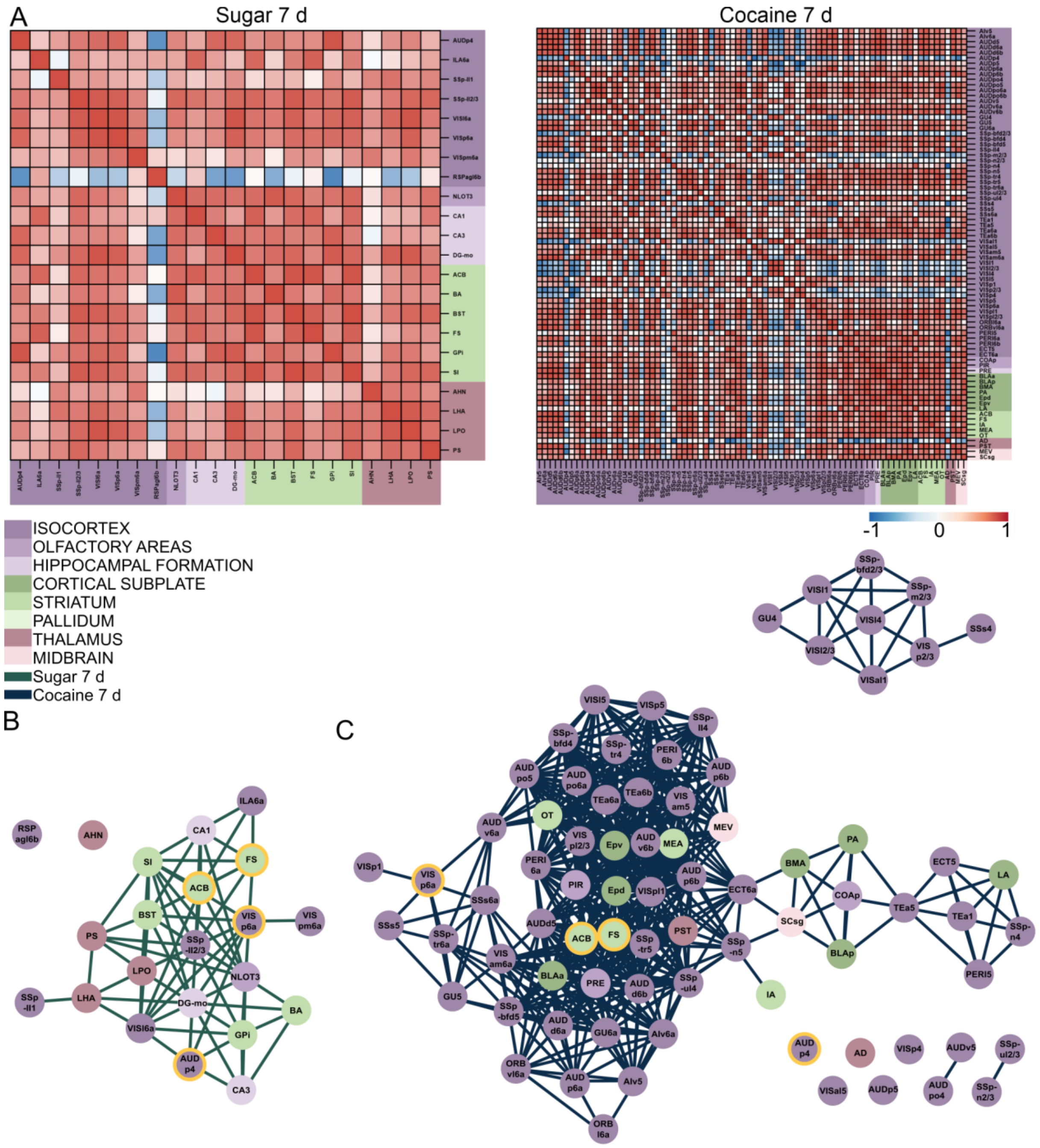

Fig. Supp. 5: Brain region correlations in structures with the highest probability of activation upon prolonged exposure to sugar or cocaine.

A) Correlation map for groups treated with natural (sugar 7 d) and pharmacological (cocaine 7 d) rewards. Color-coded anatomical annotations are depicted below the heat maps. Colors of heatmaps indicate the correlation strength between single structures, in which warmer colors indicate more similar signal densities between two structures in all mice. **B, C**) Graph Networks based on the correlation matrices of structures activated by 7 days of sugar (B, green) or cocaine (C, blue) exposure. Each dot represents a single structure and lines represent a positive correlation between two structures. Color-coded anatomical annotations correspond to these on correlation matrices on panel A. Distance and position between nodes were automatically determined by the hierarchical repulsion model. Four structures, which were activated by both rewards (the fundus of the striatum (FS), the nucleus accumbens (ACB), the primary visual area layer 6a (VISp6a), the primary auditory area layer 4

(AUDp4)) were additionally contoured with yellow color. N Water=7, N Sugar 1 d=5, N Sugar 7 d=7, N Saline=7, N Cocaine 1 d=6, N Cocaine 7 d=6. For network graph r>0.75.

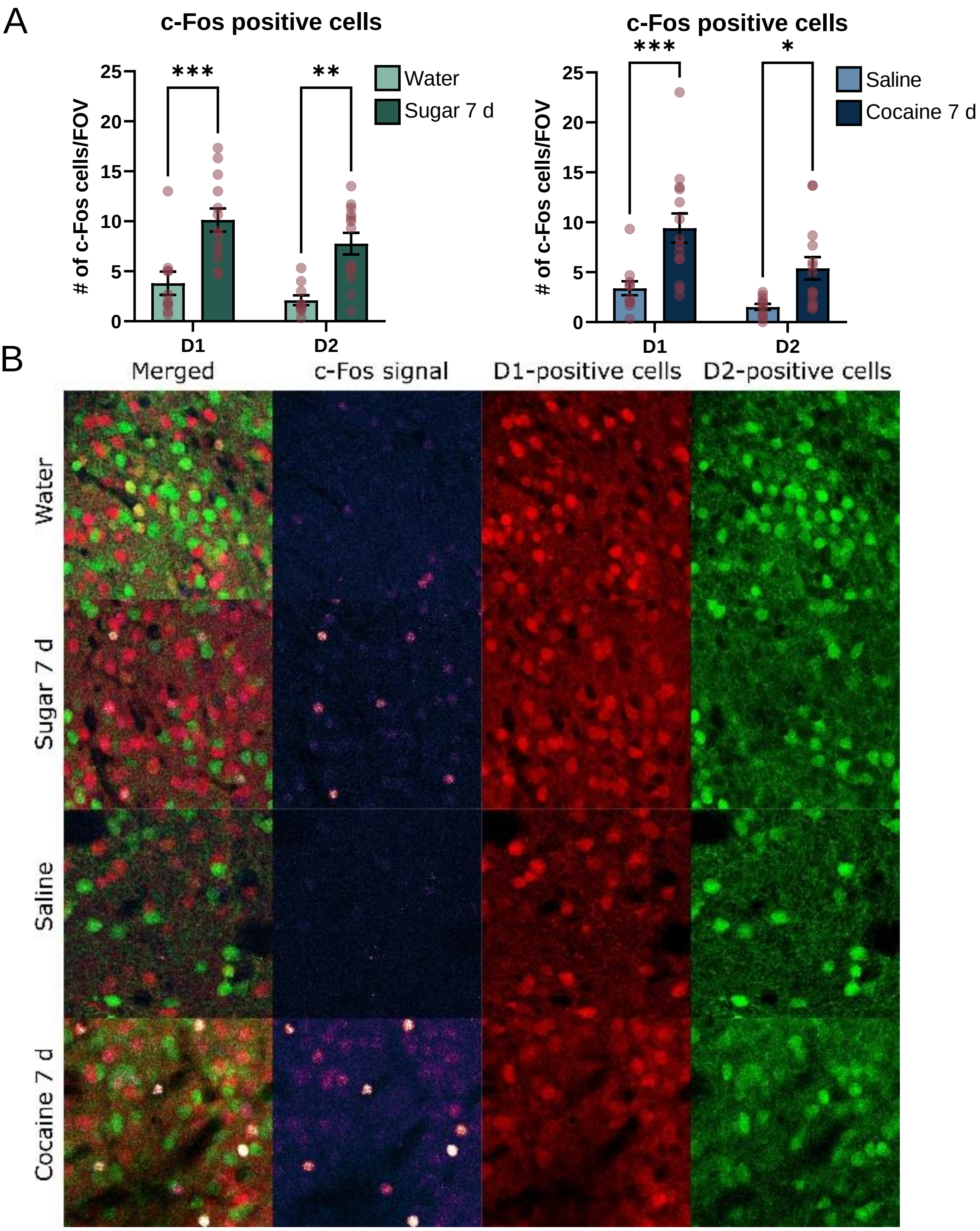

Fig. Supp. 6: c-Fos distribution in D1- and D2-positive cells in Nucleus Accumbens.

A) Number of c-Fos positive cells in D1- and D2-positive cells in Nucleus Accumbens after natural (green) and addictive treatment (blue). **B**) Exemplary images of c-Fos (HiLo) signal in D1- (red) and D2-positive (green) cells. Scale bar=40 µm.

For c-Fos N Water=10(28); N Sugar 7 d=13(38); N Saline = 11(33), N Cocaine 7 d=14(40), where N=Number of mice

(Number of planes). P–values were determined with the Two-way ANOVA test. *=p<0.05; **=p<0.01; ***=p<0.001. For c-Fos p–value D1 Water vs. D1 Sugar 7 d=0.0003; D2 Water vs. D2 Sugar 7 d=0.001; D1 Saline vs. D1 Cocaine 7 d=0.0007; D2 Saline vs. D2 Cocaine 7 d=0.0326.
